# Modelling and investigating memory immune responses in infectious disease. Application to influenza a virus and sars-cov-2 reinfections

**DOI:** 10.1016/j.idm.2024.09.009

**Published:** 2024-10-09

**Authors:** Mathilde Massard, Bruno Saussereau, Catherine Chirouze, Quentin Lepiller, Raluca Eftimie, Antoine Perasso

**Affiliations:** a Laboratoire de Mathématiques de Besançon, Université de Franche-Comté, UMR-CNRS 6623, 16 route de GRAY, 25030, Besançon, France; b Laboratoire Chrono-environnement, Université de Franche-Comté, UMR-CNRS 6249, 16 route de GRAY, 25030, Besançon, France; c Centre-Hospitalier Universitaire de Besançon, Boulevard A. Fleming, Besançon, France

**Keywords:** Within-host model, Immunology, Covid-19, SARS-CoV-2, Influenza a, Memory cells

## Abstract

Understanding effector and memory immune responses against influenza A virus (IAV) and severe acute respiratory syndrome coronavirus 2 (SARS-CoV-2) infections and re-infections is extremely important, given that they are now endemic in the community. The goal of this study is to investigate the role of memory cells and antibodies in the immune responses against IAV and SARS-CoV-2 re-infections. To this end, we adapt a previously-published within-host mathematical model (Sadria & Layton, 2021) for the primary immune response against SARS-CoV-2 infections, by including two types of memory immune cells, i.e., memory CD8^+^ T-cells and memory B-cells, and by parametrising the new model with values specific to the two viruses. We first investigate the long-term dynamics of the model by identifying the virus-free steady states and studying the conditions that ensure the stability of these states. Then, we investigate the transient dynamics of this in-host model by simulating different viral reinfection times: 20 days, 60 days and 400 days after the first encounter with the pathogen. This allows us to highlight which memory immune components have the greatest impact on the viral elimination depending on the time of reinfection. Our results suggest that memory immune responses have a greater impact in the case of IAV infections compared to SARS-CoV-2 infections. Moreover, we observe that the immune response after a secondary infection is more efficient when the reinfection occurs at a shorter time.

## 1 Introduction

COVID-19 (coronavirus disease 2019) is a viral infectious disease caused by the SARS-CoV-2. This coronavirus was discovered in December 2019 in the city of Wuhan in China (Tang et al., 2020; Zhou et al., 2020). The most common symptoms are fever, cough, fatigue and difficulty breathing. In the most severe forms, the acute respiratory distress syndrome can lead to death. Different factors may contribute to the heterogeneity of immune responses against SARS-CoV-2. These factors are age-related, genetic, sex-related, comorbidity-related (quality of the immune response influenced by arterial hypertension, diabetes, obesity, cardiovascular diseases) (de Carvalho Sales-Peres et al., 2020; Gupta, Marzook, & Ahmad, 2023; Karachaliou et al., 2021; Ursin, Shapiro, & Klein, 2020) and also related to pre-existing immunity (previous infections) (Mazzoni et al., 2021). Influenza A is a viral infectious disease caused by the influenza A virus which has several subtypes (H1N1 for example). It is a highly contagious seasonal respiratory infection that affects several million people in France each year. Influenza A infection can lead to serious forms with cases of acute respiratory distress syndrome (Jaber, Conseil, Coisel, Jung, & Chanques, 2010). Both SARS-CoV-2 and IAV (as well as other viruses) persist in the community, and people can be re-infected. Therefore, it is important to understand the within-host conditions that allow for such re-infections.

The immune system is subdivided into two main subsystems, the innate system and the adaptive system (Dempsey, Vaidya, & Cheng, 2003). Cells of the adaptive immune system include antibody-producing cells, the B-cells, which arise in the bone marrow and the effectors of cellular immune responses, the T-cells, which mature in the thymus (Bonilla & Oettgen, 2010). *CD*8^+^ T-cells are able to recognize and destroy infected cells. This cellular response, in association with the production of specific antibodies, contributes to the healing of infected patients (Bertholom, 2021; Netea et al., 2020). Adaptive immune responses are activated under the influence of signals from the innate immune system provided either directly by circulating pathogens or indirectly by antigen-presenting cells (APC) migrating to lymph nodes (Bonilla & Oettgen, 2010). A principal feature of adaptive immunity is the generation of immunological memory. During the first encounter with an antigen (pathogen), long-lived memory *CD*8^+^ T-cells and long-lived memory B-cells are established. In subsequent encounters with the same pathogen, the memory cells are quickly activated to yield a more robust protective response (Natoli & Ostuni, 2019).

To investigate the immune response against SARS-CoV-2 infection, researchers have focused their attention on various mathematical within-host models. The great majority of these models focus on first infections and model.

(i) single viral infections, usually using simplified assumptions of the immune response to this virus (Carruthers, Xu, Finnie, & Hall, 2022; Elbaz, Sohaly, & El-Metwally, 2022; Li, Xu, Liu, & Zhou, 2020; Ul Haq, Yavuz, Ali, & Ali, 2022; Xu, Carruthers, Finnie, & Hall, 2023), there are also a few complex models which include more complex immune responses (Carruthers et al., 2022; Chatterjee, Singh Sandhu, & Dixit, 2022; Ghosh, 2021; Mondal, Samui, & Chatterjee, 2022).
(ii) co-infections between SARS-CoV-2 and other pathogens: e.g., SARS-CoV-2/HIV, SARS-CoV-2/Influenza A virus, SARS-CoV-2/Plasmodium spp (Malaria), SARS-CoV-2/*Mycobacterium tuberculosis* (Tuberculosis) (Agha & Elaiw, 2022; Ahmed et al., 2022, 2023; Elaiw, Agha, Azoz, & Ramadan, 2022).

To our knowledge, until now only a few mathematical in-host models have been developed to investigate the role of memory immune responses to multiple viral infections (Cao et al., 2015; Yan, Zaloumis, Simpson, & McCaw, 2019; Zarnitsyna et al., 2016) (although there are various SIR-type models for epidemiological effects of re-infection (Atifa, Khan, Iskakova, Al-Duais, & Ahmad, 2022; McMahon & Robb, 2020).) Schuh et al. (Schuh, Markov, Veliov, & Stilianakis, 2024) propose an in-host model for SARS-CoV-2 reinfections. This model gives results about a global immune response whose information is grouped under a single compartment and not particularly on every component of the immune system (B-cells, antibodies,etc.). Xu and al (Xu, Wei, Zhang, & Demongeot, 2023). propose an in-host model to investigate the immune response focusing mainly on antibodies and interferons. The results emphasize the role of antibodies against reinfections. In contrast to these previous models, which do not consider immune responses described by both T-cells and B-cells, here we take into account these detailed immune responses. However, to balance the complexity of the various immunological interactions, we decided to focus only on simpler temporal dynamics and ignore more complex spatio-temporal dynamics (as considered in (Amoddeo, 2023)). Influenza A within-host models also focus a lot on single infections (Hancioglu, Swigon, & Clermont, 2007; Handel & Antia, 2008). Few studies also take into account long lived plasma cells (Lee et al., 2009). An overview of the different mathematical models for IAV infection is presented in (Boianelli et al., 2015).

**Fig. 1:**
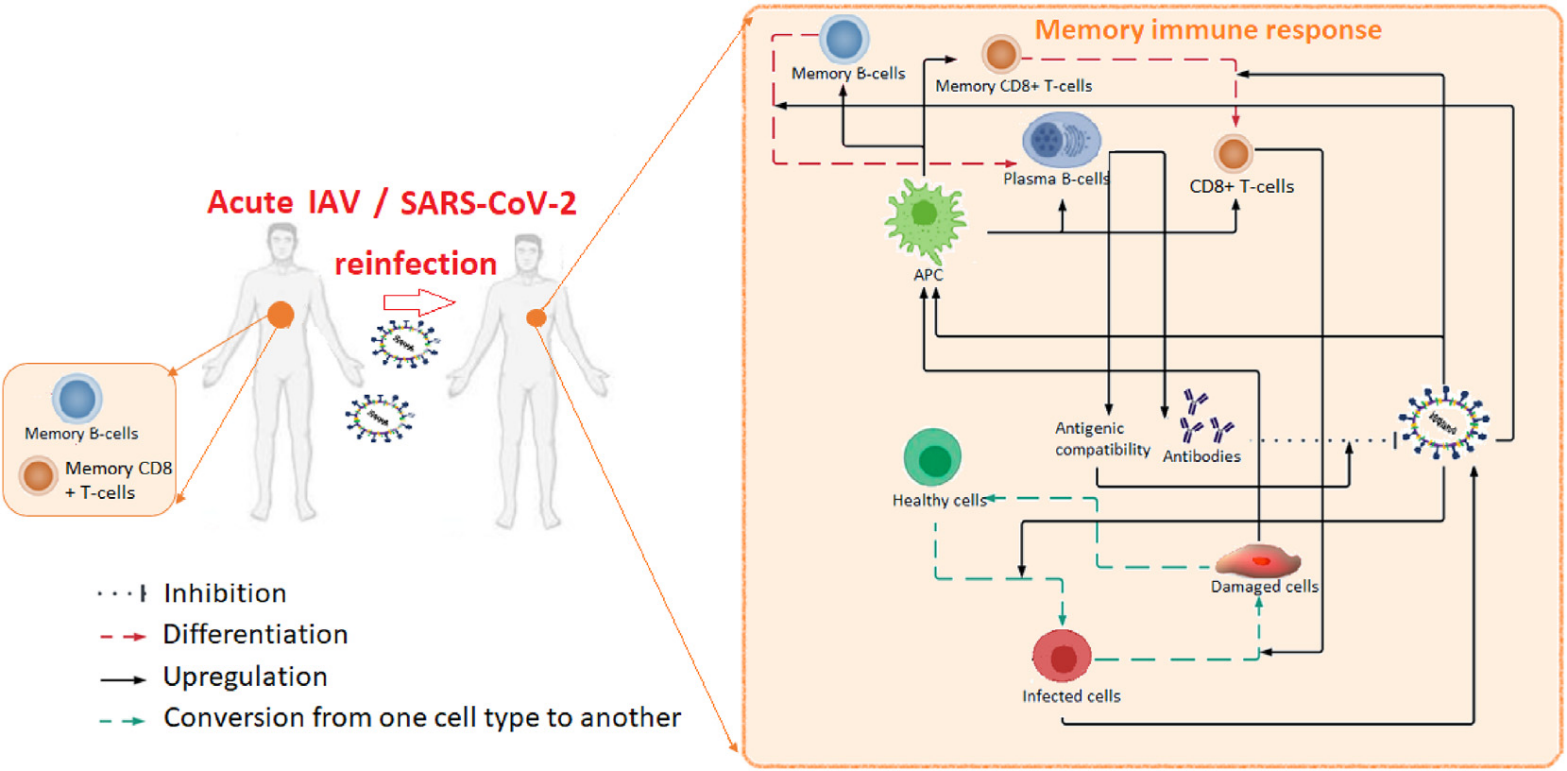
Diagram of the model (1). Interactions with memory cells are highlighted in red.

The main aim of this paper is to propose a new in-host mathematical model that would allow us to gain better understanding of the role of memory T-cells and B-cells during reinfection in infectious disease. To this end, we start with the in-host model in (Sadria & Layton, 2021) that describes the anti-viral immune response for an average individual, and adapt it to include the formation of immune memory and the activation of this memory during re-infection. Thus, the new model includes two types of memory cells, memory CD8^+^ T-cells and memory B-cells (see Fig. 1). We add terms in the equations for effector cells, plasma cells and viral load that depend on *t*∗, the time at which there is reinfection. In addition, we ignore the latent cells considered in (Sadria & Layton, 2021), and we transform a term in the equation for the effector cells to ensure that no such cells persist in the absence of infections. We ignore gamma inteferons to remove a redundancy in the modelling of anti-viral immune response observed in (Sadria & Layton, 2021): since gamma interferons are secreted mainly by CD8^+^ T-cells (Kambayashi, Assarsson, Lukacher, Ljunggren, & Jensen, 2003; Nicolet et al., 2020), including them both into the model leads to redundant anti-viral response. We therefore also ignore the resistant cells associated with this interferons.

The paper is structured as follows. In section 2 we introduce the new model. In Section 3, we investigate asymptotic dynamics through the calculation of the virus free steady states and their stability and calculate the *R*_0_. Finally, We investigate transient dynamics of the model through numerical simulations.

## 2 Materials and methods

### 2.1 Model description

In the following we present in detail a new model which describes the response of immune system to an infectious disease during secondary infections. To this end, we start with an in-host mathematical model introduced in (Sadria & Layton, 2021) for primary infections, and adapt it to consider also memory CD8^+^ T-cell and memory B-cell immune responses that develop following viral re-infections. Thus, while the model in (Sadria & Layton, 2021) focused on the interactions between virus particles, effector CD8^+^ T-cells, plasma B-cells, interferons, antibodies, and epithelial cells (that can be healthy, infected, latent infected, resistant and damaged), in our study we consider also interactions with memory CD8^+^ T-cells and memory B-cells.

● Memory CD8^+^ T-cells mediate rapid clearance of pathogens upon secondary infection owing to their elevated frequency, ready localization to peripheral sites of infection and their ability to rapidly expand and mount effector functions. Such potent long-lasting protective memory CD8^+^ T-cells develop in infections where infected-cells are effectively cleared (Ahmed et al.).
● Memory B-cells memorize the properties of the antigen in order to rapidly differentiate into plasma cells if a second infection with the same antigen occurs and thus create a more intense and more specific immune response (Anaya, Shoenfeld, Rojas-Villarraga, Levy, & Cervera, 2013).

Moreover, compared to the in-host model introduced in (Sadria & Layton, 2021), here we incorporate four modifications other than the addition of the two equations concerning the memory cells: (i) To simplify an already complex model, we have decided to ignore the latent cells since they are not part of the main memory immune response, which is the focus of this study, (ii) we replace the 1 − *E* term in the equation for effector cells in (Sadria & Layton, 2021) with a term −*E*, because effector cells must disappear when there is no virus (and a logistic-like term allows for cell persistence), (iii) we no longer consider the interactions with interferons and associated resistant cells because there is a redundancy in the modelling of anti-viral immune response: since gamma interferons are secreted mainly by CD8^+^ T-cells (Kambayashi et al., 2003; Nicolet et al., 2020), including them both into the model leads to redundant anti-viral response, (iv) we add terms which correspond to the differentiation of memory cells and reinfection in the equations for effector cells, plasma cells and viral load that depend on *t*∗, the time at which there is reinfection. Note that these assumptions are appropriate for modelling infections, where memory immune responses can clear the pathogen following secondary infections (Kalia, Sarkar, & Ahmed, 2010). Since we do not have yet enough knowledge about the role of memory immune responses in chronic infections (which are usually characterised by immune dysfunction (Kalia et al., 2010)), we do not consider such chronic infections in this study.

The variables in this new model are: the concentration of healthy epithelial cells (*H*), the concentration of infected cells (*I*), the concentration of antigen-presenting cells (*M*), damaged and dead cells (*D*), the concentration of effector CD8^+^ T-cells (*E*) and memory CD8^+^ T-cells (*T*_*m*_), the concentration of plasma B-cells (*P*) and memory B-cells (*B*_*m*_), the concentration of viral load (*V*), and the concentration of antibodies (*A*) characterized by their specificity (*S*). Note that in (Sadria & Layton, 2021) the authors have used the variable *D* to describe both damaged and dead cells, leading to the assumption incorporated into the model that these cells can provide signals that determine surrounding cells to proliferate (Brock et al., 2019). The time of reinfection is described by *t*∗ and *C* is the viral load received during reinfection at *t*∗. The interactions between all the variables are depicted in Fig. 1 and described by the system below:

(1a) $$\frac{dH}{dt}=-\gamma_{HV}VH+b_{HD}DH-a_{H}H$$

(1b) $$\frac{dI}{dt}=\gamma_{HV}VH-b_{IE}EI-a_{I}I$$

(1c) $$\frac{dM}{dt}=\left(b_{MD}D+b_{MV}V\right)\left(1-M\right)-a_{M}M$$

(1d) $$\frac{dT_{m}}{dt}=b_{TM}M\left(1-T_{m}\right)-a_{T}T_{m}+\left(-\gamma_{VT}T_{m}V\right)1_{\left[t^{*},\right)\infty [}\left(t\right)$$

(1e) $$\frac{dE}{dt}=b_{EM}ME-b_{EI}IE-a_{E}E+\left(\gamma_{VT}T_{m}V\right)1_{[t^{*},\infty [}\left(t\right)$$

(1f) $$\frac{dB_{m}}{dt}=b_{BM}M\left(1-B_{m}\right)-a_{B}B_{m}+\left(-\gamma_{VB}B_{m}V\right)1_{[t^{*},\infty [}\left(t\right)$$

(1g) $$\frac{dP}{dt}=b_{PM}MP+a_{p}\left(1-P\right)+\left(\gamma_{VB}B_{m}V\right)1_{[t^{*},\infty [}\left(t\right)$$

(1h) $$\frac{dV}{dt}=\gamma_{V}I-\gamma_{VA}SAV-\gamma_{VH}VH-\alpha_{V}V+C1_{[t^{*};t^{*}+\delta [}\left(t\right)$$

(1i) $$\frac{dA}{dt}=b_{A}P-\gamma_{AV}SAV-a_{A}A$$

(1j) $$\frac{dS}{dt}=rP\left(1-S\right)$$

(1k) D=1−H−I

In the following we describe in more details the terms contained in these equations.

Eq. (1a) describes the dynamics of healthy epithelial cells. The population of epithelial healthy cells decreases when a virus infects them due to the lytic impact of the virus (Ryu & Shin, 2021). Healthy cells are generated as a result of proliferation of healthy cells. We assume, as in (Sadria & Layton, 2021), that tissue damage induces the production of new healthy cells due to the proliferation of the remaining healthy cells (that react to apoptotic bodies from dying cells (Brock et al., 2019)). The population of healthy epithelials cells decreases also by natural decay.

Eq. (1b) describes the dynamics of epithelial infected cells. Healthy cells become infected when they meet the virus. Effector cells play direct roles in elimination of pathogens by killing infected target cells (Bonilla & Oettgen, 2010). The population of infected cells decreases also by natural decay.

Eq. (1c) describes the dynamics of APC. Damaged cells and viruses stimulate the activation of APC (Kono & Rock, 2008). The population of APC decreases by natural decay.

Eq. (1d) describes the dynamics of memory CD8^+^ T-cells. Memory T-cells are produced following a complex process of differentiation that starts with antigen presentation by APCs (Kaech, Wherry, & Ahmed, 2002). Since in (Van de Sandt, Kreijtz, & Rimmelzwaan, 2012) it was shown that the secondary encounter with the virus leads to fast proliferation of memory cells, we have decided to consider here only the role of APCs. Memory T-cells differentiate into effector cells following viral reinfection at time *t* = *t*∗ (Bonilla & Oettgen, 2010; Sette & Crotty, 2022). The population of memory T-cells decreases by natural decay.

Eq. (1e) describes the dynamics of effector CD8^+^ T-cells. APC stimulate the production of effector cells. The population of effector cells decreases when some effector cells are lost during the destruction of infected cells and also by a natural regulation of effector cells. Memory T-cells differentiate into effector cells when cells are activated following viral reinfection at time *t* = *t*∗ (Bonilla & Oettgen, 2010).

Eq. (1f) describes the dynamics of memory B-cells. Memory B-cells are produced following stimulation by APC (Hartley et al., 2020) and differentiate into plasma cells following viral reinfection at time *t*∗ (Bonilla & Oettgen, 2010). The population of memory B-cells decreases by natural decay.

Eq. (1g) describes the dynamics of plasma cells. Plasma cells develop under the direction of signals received by APC (Xu & Banchereau, 2014). Memory B-cells differentiate into plasma cells when cells are activated following viral reinfection at time *t*∗ (Bonilla & Oettgen, 2010). There is a natural decay rate of plasma cells.

Eq. (1h) describes the dynamics of viral load. Viruses replicate in infected cells, increasing viral load (Julkunen et al., 2000; Tamura, Tanimoto, & Kurata, 2005). Viral load decreases when viruses are cleared by antibodies, when the virus enters and kills healthy cells. A new viral load C appears during reinfection at time *t*∗.

Eq. (1i) describes the dynamics of antibodies. Antibodies are produced by plasma cells (Bonilla & Oettgen, 2010; Zuiani & Wesemann, 2022). The population of antibodies decreases after their fixation to the virus, or due to their clearance.

Eq. (1j) describes antibody specificity (which quantifies the affinity between antibodies and viruses). This specificity, which is assumed to be between 0 (no specificity) and 1 (full specificity), increases as plasma cells produce antibodies due to the affinity maturation process.

Eq. (1k), damaged cells serve as a marker for tissue damage (see (Hancioglu et al., 2007; Hayden et al., 1998)).

We note that a certain number of choices have been made to simplify the modeling of the immune system. For example, we assumed that cells and virions populations were uniformly distributed across the epithelial layer at all times. Also, we did not consider the role of any *CD*4 T-cells.

### 2.2 Rescaling of variables and parameters description

The basic unit of all components is $M=mol.L^{-1}$. Each component is rescaled by its homeostatic value (also in M) found in (Bocharov & Romanyukha, 1994), noted with ”∗” (See Table 1).

**Table 1:** Variables involved in model (1) and their scaling. Note that $M=mol.L^{-1}$.

Variables	Significations	Rescaling factors [Range]
*H*	Proportion of healthy cells	$H^{*}=1.7.10^{-11}\;M\;\left[1.7.10^{-12},1.7.10^{-11}\right]$
*I*	Proportion of infected cells	$H^{*}=1.7.10^{-11}\;M\;\left[1.7.10^{-12},1.7.10^{-11}\right]$
*M*	APC per homeostatic level	$M^{*}=10^{-15}\;M\;\left[5.10^{-16},3.10^{-15}\right]$
*T*_*m*_	Memory *CD*8^+^ T-cells per homeostatic level	$E^{*}=10^{-16}\;M\;\left[10^{-16},10^{-15}\right]$
*E*	Effector *CD*8^+^ T-cells per homeostatic level	$E^{*}=10^{-16}\;M\;\left[10^{-16},10^{-15}\right]$
*B*_*m*_	Memory B-cells per homeostatic level	$P^{*}=1.8.10^{-20}\;M\;\left[3.10^{-21},7.10^{-20}\right]$
*P*	Plasma B-cells per homeostatic level	$P^{*}=1.8.10^{-20}\;M\;\left[3.10^{-21},7.10^{-20}\right]$
*V*	Viral load per epithelial cells	$H^{*}=1.7.10^{-11}\;M\;\left[1.7.10^{-12},1.7.10^{-11}\right]$
*A*	Antibodies per homeostatic level	$A^{*}=8.5.10^{-13}\;M\;\left[1.7.10^{-13},1.7.10^{-12}\right]$
*D*	Proportion of damaged cells	*D* = 1 − *H* − *I*
*S*	Ab Specificity	–

In the case of epithelial cells (that can be healthy, infected, latent infected and damaged), they are rescaled compared to *H*∗, the homeostatic value of the concentration of healthy epithelial cells in LN. The concentration of the virus in any compartment is rescaled by *H*∗ too. This rescaling, proposed by Hancioglu in (Hancioglu et al., 2007) allows us to obtain parameters in *day*^−1^.

#### 2.2.1 Remark

1 mol.L^−1^ = 6∗10^20^ *particles*.*mL*^−1^ (Marchuk, Petrov, Romanyukha, & Bocharov, 1991) thus *V* = 1 in our model (after rescaling by *H*∗) corresponds to 1.2∗10^10^ *particles*.*mL*^−1^.

#### 2.2.2 IAV infection

Parameters values were taken mostly from Bocharov and Romanyukha (Bocharov & Romanyukha, 1994) and the rescaling of these values to match our units model parameters (*day*^−1^) are available in Appendix A. See Table 2 for the other references.

**Table 2:** Parameters involved in model (1) for simulation of the immune response in influenza A and SARS-CoV-2 infections. Units of all parameters: *day*^−1^.

Symbol	Biological interpretation	Value IAV [Ref]	Value SARS-CoV-2 [Ref]
*a*_*H*_	Natural death rate of healthy epithelial cells	10^−3^ (Lee et al., 2009)	10^−3^ (Lee et al., 2009)
*γ*_*V*_	Viral production rate by infected cells	400 (Bocharov & Romanyukha, 1994)	250 (Li et al., 2020)
*γ*_*VA*_	Rate of viral elimination by antibodies	146.2 (Bocharov & Romanyukha, 1994)	146.2 (Bocharov & Romanyukha, 1994)
*γ*_*VH*_	Rate of virus entry into healthy cells	1.7.10^−3^ (Bocharov & Romanyukha, 1994)	0.0001 (Chatterjee et al., 2022)
*α*_*V*_	Virus degradation/elimination rate	2 (Bocharov & Romanyukha, 1994)	1.75 (Fatehi, Bingham, Dykeman, Stockley, & Twarock, 2021)
*b*_*HD*_	Regeneration rate of epithelial cells	3 (Bocharov & Romanyukha, 1994)	3 (Bocharov & Romanyukha, 1994)
*γ*_*HV*_	Rate of infection of cells by virus	0.032 (Smith & Perelson, 2011)	0.033 (Li et al., 2020)
*b*_*IE*_	Infected cells damaged by effector cells	0.0017 (Bocharov & Romanyukha, 1994)	0.0739 (Fatehi et al., 2021)
*a*_*I*_	Infected cells death rate	1.2 (Lee et al., 2009)	0.93 (Kim et al., 2021)
*b*_*MD*_	Stimulation rate of APC by damaged cells	0.0017 Estimated	0.0017 Estimated
*b*_*MV*_	Stimulation rate of APC by virus	0.04 (Bocharov & Romanyukha, 1994)	0.4873 (Dogra et al., 2023)
*a*_*M*_	Antigen presenting cell natural death	1 (Bocharov & Romanyukha, 1994)	1 (Bocharov & Romanyukha, 1994)
*b*_*EM*_	Stimulation rate of effector cells by APC	10 (Bocharov & Romanyukha, 1994)	10 (Bocharov & Romanyukha, 1994)
*b*_*EI*_	Death of effector cells by infected cells	10^−8^ (Bocharov & Romanyukha, 1994)	10^−8^ (Bocharov & Romanyukha, 1994)
*a*_*E*_	Effector cell natural death rate	0.4 (Bocharov & Romanyukha, 1994)	0.4 (Bocharov & Romanyukha, 1994)
*b*_*PM*_	Plasma cell production rate	10 (Bocharov & Romanyukha, 1994)	10 (Bocharov & Romanyukha, 1994)
*a*_*P*_	Plasma cell natural death rate	0.4 (Bocharov & Romanyukha, 1994)	0.2 (Ghosh, 2021)
*b*_*A*_	Antibody production rate per plasma cell	0.8 (Lee et al., 2009)	1 (Ghosh, 2021)
*r*	Change in antibody specificity	3∗10^−2^ Estimated	3∗10^−2^ Estimated
*γ*_*AV*_	Rate at which antibodies bind to viruses	146 (Bocharov & Romanyukha, 1994)	146 (Bocharov & Romanyukha, 1994)
*a*_*A*_	Antibody natural death rate	0.043 (Bocharov & Romanyukha, 1994)	0.07 (Ghosh, 2021)
*b*_*BM*_	Memory B-cells production rate	15 Estimated	15 Estimated
*γ*_*VB*_	Rate of the differentiation of memory B-cells	0.2 (BC centre for Disease Control, 2009)	0.2 (BC centre for Disease Control, 2009)
*b*_*TM*_	Memory CD8^+^ T-cells production rate	15 Estimated	15 Estimated
*γ*_*VT*_	Rate of the differentiation of memory T-cells	0.2 (BC centre for Disease Control, 2009)	0.2 (BC centre for Disease Control, 2009)
*a*_*B*_	Memory B-cells natural death rate	0.004 (Dan et al., 2021)	0.004 (Dan et al., 2021)
*a*_*T*_	Memory CD8^+^ T-cells natural death rate	0.01 (Zarnitsyna et al., 2016)	0.01 (Dan et al., 2021)

#### 2.2.3 SARS-CoV-2 infection

Note that the parameters in (Sadria & Layton, 2021) were taken mostly from the literature for influenza A because in 2021 few data was available about the immune response in SARS-CoV-2 infection. We therefore do not use these data and will take our values from the literature for SARS-CoV-2. See references in Table 2.

Some values are the same regardless of the infection, so they will have the same value and the same reference in Table 2.

## 3 Results

Model (1) can be written as follows:

$$\frac{dx\left(t\right)}{dt}=f\left(x\left(t\right)\right)+1_{t\geq t^{*}}g\left(x\left(t\right)\right)+1_{[t^{*},t^{*}+\delta [}h\left(x\left(t\right)\right),$$

with,

$$\begin{array}{ll} x\left(t\right) & =\;{\left(H,I,M,T_{m},E,B_{m},P,V,A,S\right)}^{T}\\ f\left(x\left(t\right)\right) & =\;\left(-\gamma_{HV}VH+b_{HD}DH-a_{H}H,\gamma_{HV}VH-b_{IE}EI-a_{I}I,\right)\\ & \left(b_{MD}D+b_{MV}V\right)\left(1-M\right)-a_{M}M,b_{TM}M\left(1-T_{m}\right)-a_{T}T_{m},b_{EM}ME-b_{EI}IE-a_{E}E,\\ & b_{BM}M\left(1-B_{m}\right)-a_{B}B_{m},b_{PM}MP+a_{P}\left(1-P\right),\gamma_{V}I-\gamma_{VA}SAV-\gamma_{VH}VH-\alpha_{V}V,\\ & {\left(b_{A}P-\gamma_{AV}SAV-a_{A}A,rP\left(1-S\right)\right)}^{T}\\ g\left(x\left(t\right)\right) & ={\left(0,0,0,-\gamma_{VT}T_{m}V,\gamma_{VT}T_{m}V,-\gamma_{VB}B_{m}V,\gamma_{VB}B_{m}V,0,0,0\right)}^{T}\\ h\left(x\left(t\right)\right) & ={\left(0,0,0,0,0,0,0,C,0,0\right)}^{T} \end{array}$$

This non-autonomous system can be divided into 3 sub-systems which are sequences of each other:

For *t* < *t*∗,

$$\left(S_{1}\right)=\left\{\begin{array}{lll} \frac{d\bar{x}\left(t\right)}{dt} & =\;f\left(\bar{x}\left(t\right)\right) & \;\\ t & \in & \left[0,t^{*}\right) \end{array}\right)$$

For $t\in \left[\left(t^{*},t^{*}+\delta \right)\right)$,

$$\left(S_{2}\right)=\left\{\begin{array}{ll} \frac{d\tilde{x}\left(t\right)}{dt} & =\;f\left(\tilde{x}\left(t\right)\right)+g\left(\tilde{x}\left(t\right)\right)+h\left(\tilde{x}\left(t\right)\right)\\ \tilde{x}\left(t^{*}\right) & =\;\bar{x}\left(t^{*}\right) \end{array}\right)$$

For *t* ≥ *t*∗ + *δ*,

$$\left(S_{3}\right)=\left\{\begin{array}{ll} \frac{dx\left(t\right)}{dt} & =\;f\left(x\left(t\right)\right)+g\left(x\left(t\right)\right)\\ x\left(t^{*}+\delta \right) & =\;\tilde{x}\left(t^{*}+\delta \right) \end{array}\right)$$

This division allows us to carry out a general analysis of the model by considering an analysis on (*S*_3_), which is an autonomous system. Thus we can use the next generation matrix method for the calculation of the basic reproduction number *R*_0_, see subsection 3.2.

### 3.1 Virus free steady states and their local stability

We investigate the model (1) (by using (*S*_3_)) to identify all possible virus free steady states and determine their stability. The aim is to find all healthy virus-free steady states with memory cells always stable. This is important if we want to understand the biological conditions that ensures the permanent elimination of the virus and the formation of a healthy, persistent anti-viral immune response. We assume that *V* = 0, and thus we obtain two positives steady states.

1. $\left(H,I,M,T_{m},E,B_{m},P,V,A,S,D\right)=\left(H^{*},0,M^{*},T_{m}^{*},0,B_{m}^{*},P^{*},0,A^{*},1,D^{*}\right)$;
2. (*H*, *I*, *M*, *T*_*m*_, *E*, *B*_*m*_, *P*, *V*, *A*, *S*, *D*) = (0, 0, *M*_1_, *T*_*m*1_, 0, *B*_*m*1_, *P*_1_, 0, *A*_1_, 1, 1);

with,

$$\begin{array}{ll} H^{*} & =\;\frac{b_{HD}-a_{H}}{b_{HD}},\;\;\;M^{*}=\frac{b_{MD}a_{H}}{a_{M}b_{HD}+b_{MD}a_{H}},\;\;\;T_{m}^{*}=\frac{b_{TM}b_{MD}a_{H}}{a_{T}a_{M}b_{HD}+a_{T}b_{MD}a_{H}+b_{TM}b_{MD}a_{H}},\\ B_{m}^{*} & =\frac{b_{BM}b_{MD}a_{H}}{a_{B}a_{M}b_{HD}+a_{B}b_{MD}a_{H}+b_{BM}b_{MD}a_{H}},P^{*}=\frac{a_{P}\left(a_{M}b_{HD}+b_{MD}a_{H}\right)}{a_{M}a_{P}b_{HD}+a_{P}b_{MD}a_{H}-b_{MD}b_{PM}a_{H}},\\ A^{*} & =\frac{b_{A}a_{P}\left(a_{M}b_{HD}+b_{MD}a_{H}\right)}{\left(a_{M}a_{P}b_{HD}+a_{P}b_{MD}a_{H}-b_{MD}b_{PM}a_{H}\right)a_{A}}=P^{*}\frac{b_{A}}{a_{A}},\;\;\;D^{*}=\frac{a_{H}}{b_{HD}}. \end{array}$$

and,

$$\begin{array}{ll} M_{1} & =\;\frac{b_{MD}}{a_{M}+b_{MD}},\;\;T_{m1}=\frac{b_{TM}b_{MD}}{a_{T}a_{M}+a_{T}b_{MD}+b_{TM}b_{MD}},\;\;\;\;B_{m1}=\frac{b_{BM}b_{MD}}{a_{B}a_{M}+a_{B}b_{MD}+b_{BM}b_{MD}},\\ P_{1} & =\frac{a_{P}\left(a_{M}+b_{MD}\right)}{a_{M}a_{P}+a_{P}b_{MD}-b_{MD}b_{PM}},\;\;\;\;A_{1}=\frac{a_{P}b_{A}\left(a_{M}+b_{MD}\right)}{a_{A}\left(a_{M}a_{P}+a_{P}b_{MD}-b_{MD}b_{PM}\right)}. \end{array}$$

#### 3.1.1 Remark

Note that the steady states #1 gives the impression that it is possible to have a co-existence of healthy and damaged/dead cells in the absence of any infection. In fact, for the parameter values listed in Table 2, we obtain either *H* ≈ 1 and *D* ≈ 0.

We note that steady state #1 is particularly interesting because it leads to a state where a large part of the epithelial cells are healthy. There is no longer any viral load or effector cells. Investigating the stability of this steady state will allow us to understand the biological conditions that ensures the permanent elimination of the virus. Steady state v#2 is also interesting because there are no healthy cells, all cells are damaged. Looking at the conditions under which this steady state is unstable is interesting to know the conditions for not remaining in this state.

##### 3.1.1.1 E**xistence and stability of the first steady state #1**

The steady state #1 is (*H*, *I*, *M*, *T*_*m*_, *E*, *B*_*m*_, *P*, *V*, *A*, *S*, *D*) = $\left(H^{*},0,M^{*},T_{m}^{*},0,B_{m}^{*},P^{*},0,A^{*},1,D^{*}\right)$. It is biologically meaningful if *H*∗, *M*∗,$T_{m}^{*}$,$B_{m}^{*}$, *P*∗,*A*∗ and *D*∗ are positives. Thus, the following conditions must be respected:

(2) $$a_{H}<b_{HD},$$

(3) $$a_{P}\left(a_{M}b_{HD}+b_{MD}a_{H}\right)>b_{MD}b_{PM}a_{H},$$

The eigenvalues of the Jacobian evaluated at this steady state are as follows:

$$\begin{array}{ll} \lambda_{1} & =\;-b_{HD}+a_{H};\;\;\;\lambda_{2}=\frac{-\left(a_{T}a_{M}b_{HD}+a_{T}b_{MD}a_{H}+b_{TM}b_{MD}a_{H}\right)}{a_{M}b_{HD}+b_{MD}a_{H}};\\ \lambda_{3} & =\frac{-\left(a_{E}a_{M}b_{HD}+a_{E}b_{MD}a_{H}-b_{EM}b_{MD}a_{H}\right)}{a_{M}b_{HD}+b_{MD}a_{H}};\;\;\lambda_{4}=\frac{-\left(a_{B}a_{M}b_{HD}+a_{B}b_{MD}a_{H}+b_{BM}b_{MD}a_{H}\right)}{a_{M}b_{HD}+b_{MD}a_{H}};\\ \lambda_{5} & =\frac{-\left(a_{M}a_{P}b_{HD}+a_{P}b_{MD}a_{H}-b_{MD}b_{PM}a_{H}\right)}{a_{M}b_{HD}+b_{MD}a_{H}};\;\;\;\lambda_{6}=-a_{A};\\ \lambda_{7} & =\frac{-ra_{P}\left(a_{M}b_{HD}+b_{MD}a_{H}\right)}{a_{M}a_{P}b_{HD}+a_{P}b_{MD}a_{H}-b_{MD}b_{PM}a_{H}};\;\;\;\lambda_{8}=\frac{-a_{H}b_{MD}+a_{M}b_{HD}}{b_{HD}};\;\;\;\lambda_{9}=\Phi ;\;\;\;\lambda_{10}=\Psi ; \end{array}$$

with Φ and Ψ:

$\Phi =\frac{-1}{2}\left(a_{I}+\alpha_{V}+\gamma_{VH}\right)-\frac{b_{A}\gamma_{VA}a_{p}b_{HD}\left(a_{M}b_{HD}+b_{MD}a_{H}\right)}{2a_{A}b_{HD}\left(b_{MD}\left(a_{P}-b_{PM}\right)a_{H}+a_{M}a_{P}b_{HD}\right)}+\frac{\gamma_{VH}a_{H}}{2b_{HD}}$

+$\frac{1}{2}{\left({\left(a_{I}-\alpha_{V}-\gamma_{VH}-\frac{b_{A}\gamma_{VA}a_{p}b_{HD}\left(a_{M}b_{HD}+b_{MD}a_{H}\right)}{a_{A}b_{HD}\left(b_{MD}\left(a_{P}-b_{PM}\right)a_{H}+a_{M}a_{P}b_{HD}\right)}+\frac{\gamma_{VH}a_{H}}{b_{HD}}\right)}^{2}+4\gamma_{HV}\gamma_{V}\left(1-\frac{a_{H}}{b_{HD}}\right)\right)}^{1/2}$,

$\Psi =\frac{-1}{2}\left(a_{I}+\alpha_{V}+\gamma_{VH}\right)-\frac{b_{A}\gamma_{VA}a_{p}b_{HD}\left(a_{M}b_{HD}+b_{MD}a_{H}\right)}{2a_{A}b_{HD}\left(b_{MD}\left(a_{P}-b_{PM}\right)a_{H}+a_{M}a_{P}b_{HD}\right)}+\frac{\gamma_{VH}a_{H}}{2b_{HD}}$

$-\frac{1}{2}{\left({\left(a_{I}-\alpha_{V}-\gamma_{VH}-\frac{b_{A}\gamma_{VA}a_{p}b_{HD}\left(a_{M}b_{HD}+b_{MD}a_{H}\right)}{a_{A}b_{HD}\left(b_{MD}\left(a_{P}-b_{PM}\right)a_{H}+a_{M}a_{P}b_{HD}\right)}+\frac{\gamma_{VH}a_{H}}{b_{HD}}\right)}^{2}+4\gamma_{HV}\gamma_{V}\left(1-\frac{a_{H}}{b_{HD}}\right)\right)}^{1/2}$,

We have *b*_*HD*_ > *a*_*H*_, see (2), which is equivalent to $1-\frac{a_{H}}{b_{HD}}>0$ so the roots.

${\left({\left(a_{I}-\alpha_{V}-\gamma_{VH}-\frac{b_{A}\gamma_{VA}a_{p}b_{HD}\left(a_{M}b_{HD}+b_{MD}a_{H}\right)}{a_{A}b_{HD}\left(b_{MD}\left(a_{P}-b_{PM}\right)a_{H}+a_{M}a_{P}b_{HD}\right)}+\frac{\gamma_{VH}a_{H}}{b_{HD}}\right)}^{2}+4\gamma_{HV}\gamma_{V}\left(1-\frac{a_{H}}{b_{HD}}\right)\right)}^{1/2}$ and.

${\left({\left(a_{I}-\alpha_{V}-\gamma_{VH}-\frac{b_{A}\gamma_{VA}a_{p}b_{HD}\left(a_{M}b_{HD}+b_{MD}a_{H}\right)}{a_{A}b_{HD}\left(b_{MD}\left(a_{P}-b_{PM}\right)a_{H}+a_{M}a_{P}b_{HD}\right)}+\frac{\gamma_{VH}a_{H}}{b_{HD}}\right)}^{2}+4\gamma_{HV}\gamma_{V}\left(1-\frac{a_{H}}{b_{HD}}\right)\right)}^{1/2}$ are well defined.

This steady state is always locally stable when all the eigenvalues are negative. Under the conditions where the components of the steady state are well defined (i.e. positive, see (2)) only the following three conditions remain:

Φ < 0, Ψ < 0 and *a*_*E*_ (*a*_*M*_*b*_*HD*_ + *b*_*MD*_*a*_*H*_) > *b*_*MD*_*b*_*EM*_*a*_*H*_.

Under the condition *b*_*HD*_ > *a*_*H*_, see (2), we obtain the following equivalence:

$$\begin{array}{ll} \Phi <0 & \Leftrightarrow \left(a_{I}+\alpha_{V}+\gamma_{VH}\right)+\frac{b_{A}\gamma_{VA}a_{p}b_{HD}\left(a_{M}b_{HD}+b_{MD}a_{H}\right)}{a_{A}b_{HD}\left(b_{MD}\left(a_{P}-b_{PM}\right)a_{H}+a_{M}a_{P}b_{HD}\right)}-\frac{\gamma_{VH}a_{H}}{b_{HD}}\\ & >\left({\left(a_{I}-\alpha_{V}-\gamma_{VH}-\frac{b_{A}\gamma_{VA}a_{p}b_{HD}\left(a_{M}b_{HD}+b_{MD}a_{H}\right)}{a_{A}b_{HD}\left(b_{MD}\left(a_{P}-b_{PM}\right)a_{H}+a_{M}a_{P}b_{HD}\right)}+\frac{\gamma_{VH}a_{H}}{b_{HD}}\right)}^{2}\right)\\ & \;+\;4\gamma_{HV}\gamma_{V}{\left(1-\frac{a_{H}}{b_{HD}}\right)}^{1/2}\\ & \Leftrightarrow a_{I}\left(\alpha_{V}+\gamma_{VH}+\frac{b_{A}\gamma_{VA}a_{p}b_{HD}\left(a_{M}b_{HD}+b_{MD}a_{H}\right)}{a_{A}b_{HD}\left(b_{MD}\left(a_{P}-b_{PM}\right)a_{H}+a_{M}a_{P}b_{HD}\right)}-\frac{\gamma_{VH}a_{H}}{b_{HD}}\right)\\ & >\gamma_{HV}\gamma_{V}\left(1-\frac{a_{H}}{b_{HD}}\right)\\ & \Leftrightarrow a_{I}\gamma_{VH}\left(1-\frac{a_{H}}{b_{HD}}\right)+a_{I}\left(\alpha_{V}+\frac{b_{A}\gamma_{VA}a_{p}b_{HD}\left(a_{M}b_{HD}+b_{MD}a_{H}\right)}{a_{A}b_{HD}\left(b_{MD}\left(a_{P}-b_{PM}\right)a_{H}+a_{M}a_{P}b_{HD}\right)}\right)\\ & >\gamma_{HV}\gamma_{V}\left(1-\frac{a_{H}}{b_{HD}}\right)\\ & \Leftrightarrow a_{I}\gamma_{VH}+\frac{a_{I}b_{HD}}{b_{HD}-a_{H}}\left(\alpha_{V}+\frac{b_{A}\gamma_{VA}a_{p}b_{HD}\left(a_{M}b_{HD}+b_{MD}a_{H}\right)}{a_{A}b_{HD}\left(b_{MD}\left(a_{P}-b_{PM}\right)a_{H}+a_{M}a_{P}b_{HD}\right)}\right)>\gamma_{HV}\gamma_{V} \end{array}$$

Using the basic reproduction number, see (5), we obtain then:

$$\begin{array}{ll} \Phi <0 & \Leftrightarrow \gamma_{V}\gamma_{HV}\left(\frac{1}{R_{0}^{2}}-1\right)<0\\ & \Leftrightarrow R_{0}<1 \end{array}$$

because *R*_0_ is positive. Thus, the first stability condition for the first steady state is *R*_0_ < 1. The second condition, Ψ < 0, is in fact always satisfied. Indeed,

$$\begin{array}{ll} \Psi <0 & \Leftrightarrow \;\frac{-1}{2}\left(a_{I}+\alpha_{V}+\gamma_{VH}\right)-\frac{b_{A}\gamma_{VA}a_{p}b_{HD}\left(a_{M}b_{HD}+b_{MD}a_{H}\right)}{2a_{A}b_{HD}\left(b_{MD}\left(a_{P}-b_{PM}\right)a_{H}+a_{M}a_{P}b_{HD}\right)}+\frac{\gamma_{VH}a_{H}}{2b_{HD}}\\ & \;<\frac{1}{2}\left[{\left(a_{I}-\alpha_{V}-\gamma_{VH}-\frac{b_{A}\gamma_{VA}a_{p}b_{HD}\left(a_{M}b_{HD}+b_{MD}a_{H}\right)}{a_{A}b_{HD}\left(b_{MD}\left(a_{P}-b_{PM}\right)a_{H}+a_{M}a_{P}b_{HD}\right)}+\frac{\gamma_{VH}a_{H}}{b_{HD}}\right)}^{2}\right)\\ & {\left(\;+\;4\gamma_{HV}\gamma_{V}\left(1-\frac{a_{H}}{b_{HD}}\right)\right]}^{1/2}\\ & \Leftrightarrow -a_{I}-\alpha_{V}-\gamma_{VH}-\frac{b_{A}\gamma_{VA}a_{p}b_{HD}\left(a_{M}b_{HD}+b_{MD}a_{H}\right)}{a_{A}b_{HD}\left(b_{MD}\left(a_{P}-b_{PM}\right)a_{H}+a_{M}a_{P}b_{HD}\right)}+\frac{\gamma_{VH}a_{H}}{b_{HD}}\\ & <\left[{\left(a_{I}-\alpha_{V}-\gamma_{VH}-\frac{b_{A}\gamma_{VA}a_{p}b_{HD}\left(a_{M}b_{HD}+b_{MD}a_{H}\right)}{a_{A}b_{HD}\left(b_{MD}\left(a_{P}-b_{PM}\right)a_{H}+a_{M}a_{P}b_{HD}\right)}+\frac{\gamma_{VH}a_{H}}{b_{HD}}\right)}^{2}\right)\\ & {\left(\;+4\gamma_{HV}\gamma_{V}\left(1-\frac{a_{H}}{b_{HD}}\right)\right]}^{1/2} \end{array}$$

The right handside of the inequality:

${\left({\left(a_{I}-\alpha_{V}-\gamma_{VH}-\frac{b_{A}\gamma_{VA}a_{p}b_{HD}\left(a_{M}b_{HD}+b_{MD}a_{H}\right)}{a_{A}b_{HD}\left(b_{MD}\left(a_{P}-b_{PM}\right)a_{H}+a_{M}a_{P}b_{HD}\right)}+\frac{\gamma_{VH}a_{H}}{b_{HD}}\right)}^{2}+4\gamma_{HV}\gamma_{V}\left(1-\frac{a_{H}}{b_{HD}}\right)\right)}^{1/2}$ is always positive while the left handside is always negative. Indeed $-a_{I}-\frac{b_{A}\gamma_{VA}a_{p}b_{HD}\left(a_{M}b_{HD}+b_{MD}a_{H}\right)}{a_{A}b_{HD}\left(b_{MD}\left(a_{P}-b_{PM}\right)a_{H}+a_{M}a_{P}b_{HD}\right)}$ is always negative under conditions (2) and $-\alpha_{V}-\gamma_{VH}+\frac{\gamma_{VH}a_{H}}{b_{HD}}=-\alpha_{V}+\gamma_{VH}\left(\frac{a_{H}}{b_{HD}}-1\right)$ is always negative under condition (2). Thus, Ψ < 0 is always satisfied under condition (2).

The third and last condition *a*_*E*_ (*a*_*M*_*b*_*HD*_ + *b*_*MD*_*a*_*H*_) > *b*_*MD*_*b*_*EM*_*a*_*H*_ implies that *a*_*E*_ or *a*_*H*_ or *b*_*HD*_ must be large, but biologically *a*_*E*_ and *a*_*H*_ large do not make sense, so this implies that *b*_*HD*_, the regeneration of epithelial cells, must be large enough. However, the natural death rates of effector cells (*a*_*E*_) and healthy epithelial cells (*a*_*H*_) are quite low, as recorded by various experimental studies that calculated the half-lives of such cells (McDonagh & Bell, 1995; Park et al., 2016; Peters-Hall et al., 2018). Therefore, for the parameter values used throughout this study (and listed in Table 2) the stability of this steady state requires that parameter *b*_*HD*_ is large enough (Note that large values for this parameter were also considered in (Bocharov & Romanyukha, 1994)).

To sum up, the two biological conditions that ensures the permanent elimination of the virus and the formation of a healthy, persistent anti-viral immune response is: *R*_0_ < 1 and the regeneration of epithelial cells must be large enough.

##### 3.1.1.2 E**xistence and stability of the second steady state v#2**

The steady state v#2 is (*H*, *I*, *M*, *T*_*m*_, *E*, *B*_*m*_, *P*, *V*, *A*, *S*, *D*) = (0, 0, *M*_1_, *T*_*m*1_, 0, *B*_*m*1_, *P*_1_, 0, *A*_1_, 1, 1). It is biologically meaningful if *M*_1_,*T*_*m*1_,*B*_*m*1_,*P*_1_ and *A*_1_ are positives. Thus, the following condition must be respected:

(4) $$a_{P}\left(a_{M}+b_{MD}\right)>b_{MD}b_{PM}.$$

The eigenvalues of the Jacobian evaluated at this steady state are as follows:

$$\begin{array}{ll} \lambda_{1} & =\;b_{HD}-a_{H};\;\;\lambda_{2}=-a_{I};\\ \lambda_{3} & =\frac{-a_{A}\alpha_{V}\left(a_{M}a_{P}+a_{P}b_{MD}-b_{MD}b_{PM}\right)-a_{P}b_{A}\gamma_{VA}\left(a_{M}+b_{MD}\right)}{\left(a_{M}a_{P}+a_{P}b_{MD}-b_{MD}b_{PM}\right)a_{A}};\\ \lambda_{4} & =-b_{MD}-a_{M};\;\;\lambda_{5}=\frac{-\left(a_{T}a_{M}+a_{T}b_{MD}+b_{TM}b_{MD}\right)}{a_{M}+b_{MD}};\\ \lambda_{6} & =\frac{-\left(a_{E}a_{M}+a_{E}b_{MD}-b_{EM}b_{MD}\right)}{a_{M}+b_{MD}};\\ \lambda_{7} & =\frac{-\left(a_{B}a_{M}+a_{B}b_{MD}+b_{BM}b_{MD}\right)}{a_{M}+b_{MD}};\;\;\lambda_{8}=\frac{-\left(a_{M}a_{P}+a_{P}b_{MD}-b_{MD}b_{PM}\right)}{a_{M}+b_{MD}};\;\;\lambda_{9}=-a_{A};\\ \lambda_{10} & =\frac{-ra_{P}\left(a_{M}+b_{MD}\right)}{a_{M}a_{P}+a_{P}b_{MD}-b_{MD}b_{PM}}. \end{array}$$

The steady state v#2 is locally unstable when at least one eigenvalue is positive. Under the condition where the components of this steady state are well defined (i.e. positive, see 4), only the two eigenvalues below can be positive:

*λ*_1_ = *b*_*HD*_ − *a*_*H*_ and $\lambda_{6}=\frac{-\left(a_{E}a_{M}+a_{E}b_{MD}-b_{EM}b_{MD}\right)}{a_{M}+b_{MD}}$.

The condition *λ*_1_ > 0 i.e. *b*_*HD*_ > *a*_*H*_ means that when the regeneration of epithelial cells is greater than the natural death of epithelial cells, this steady state is unstable. Thus, we can interpret that the system can approach a critical state where all these cells are damaged but if the regeneration of epithelial cells is efficient enough i.e. greater than the death of healthy epithelial cells then the system will not remain in this unhealthy state.

### 3.2 Basic reproduction number *R*_0_

The next generation matrix method allows us to calculate the basic reproduction number for autonomous systems in a finite dimensional case (See (Perasso, 2018) for more details about this number). It was described by van den Driessche and Watmough in (Van den Driessche & Watmough, 2002) and is based on the definition of *R*_0_ from Diekmann and Heesterbeek in (Diekmann, Heesterbeek, & Metz, 1990) as the dominant eigenvalue of the ”next generation matrix”. The notion of *R*_0_ extends in the case of within-host models to infected cells. Applying this method to the autonomous subsystem *S*_3_ we obtain (See Appendix B for details.):

$$R_{0}\left(x_{0}\right)={\left(\frac{\gamma_{HV}H_{0}\gamma_{V}}{A_{0}E_{0}S_{0}b_{IE}\gamma_{VA}+A_{0}S_{0}a_{I}\gamma_{VA}+E_{0}H_{0}b_{IE}\gamma_{VH}+E_{0}\alpha_{V}b_{IE}+H_{0}a_{I}\gamma_{VH}+a_{I}\alpha_{V}}\right)}^{1/2},$$

with *x*_0_ = (*H*, *I*, *M*, *T*_*m*_, *E*, *B*_*m*_, *P*, *V*, *A*, *S*, *D*) = (*H*_0_, *I*_0_, *M*_0_, *T*_*m*0_, *E*_0_, *B*_0_, *P*_0_, 0, *A*_0_, *S*_0_, *D*_0_) a virus free steady state among the steady states presented in subsection 3.1. Among these steady states, the steady state v*#*2 gives *R*_0_ (*x*_0_) = 0. Using the steady state v*#*1 we thus obtain a global *R*_0_ defined as follows:

(5) $$R_{0}={\left(\frac{\gamma_{HV}\frac{b_{HD}-a_{H}}{b_{HD}}\gamma_{V}}{\frac{b_{A}a_{P}\left(a_{M}b_{HD}+b_{MD}a_{H}\right)}{\left(a_{M}a_{P}b_{HD}+a_{P}b_{MD}a_{H}-b_{MD}b_{PM}a_{H}\right)a_{A}}a_{I}\gamma_{VA}+\frac{b_{HD}-a_{H}}{b_{HD}}a_{I}\gamma_{VH}+a_{I}\alpha_{V}}\right)}^{1/2}.$$

We can calculate the *R*_0_ which corresponds to influenza A and COVID-19 from the data in Table 2. We obtain $R_{0}^{\mathrm{IAV}}=0.0635<1$ and $R_{0}^{\mathrm{SARS-CoV -2}}=0.0728<1$.

In both cases the basic reproduction number is less than 1, which implies that in our simulations, see subsection 3.3, we will reach a steady state in a long time.

### 3.3 Numerical simulations

In this section we investigate numerically the dynamics of model (1) following viral acute reinfection: at 20, 60 and 400 days after the first infection. In 3.3.1, 3.3.2, 3.3.3 we focus on IAV reinfections, while in 3.3.4, 3.3.5, 3.3.6 we focus on SARS-CoV-2 reinfections. In section 3.3.7 we focus on the impact of varying the viral load received during reinfections. The initial conditions for all numerical simulations are: *A* = 1, *B*_*m*_ = 0, *E* = 1, *H* = 1, *I* = 0, *M* = 0, *P* = 1, *S* = 0.01, *T*_*m*_ = 0, *V* = 0.8. These values describe the situation where the components of the immune response first encounter the virus and already start reacting to it. We assume *V* = 0.8, which corresponds to 8.10^9^ *particle*/*mL* (since in (Geng & Wang, 2023) the authors have shown that an acute infection corresponds to a viral load greater than 10^8^ particle/mL).

#### 3.3.1 IAV reinfection after twenty days

**Fig. 2:**
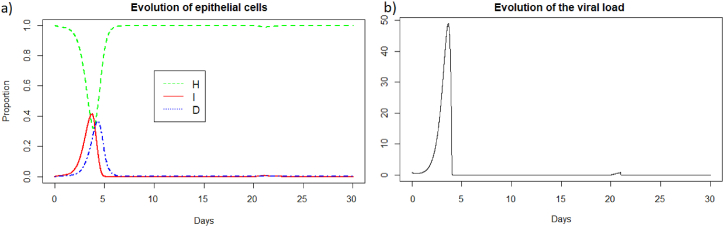
(a) Evolution of epithelial cells and (b) evolution of the viral load after IAV reinfection at t = 20 days.

**Fig. 3:**
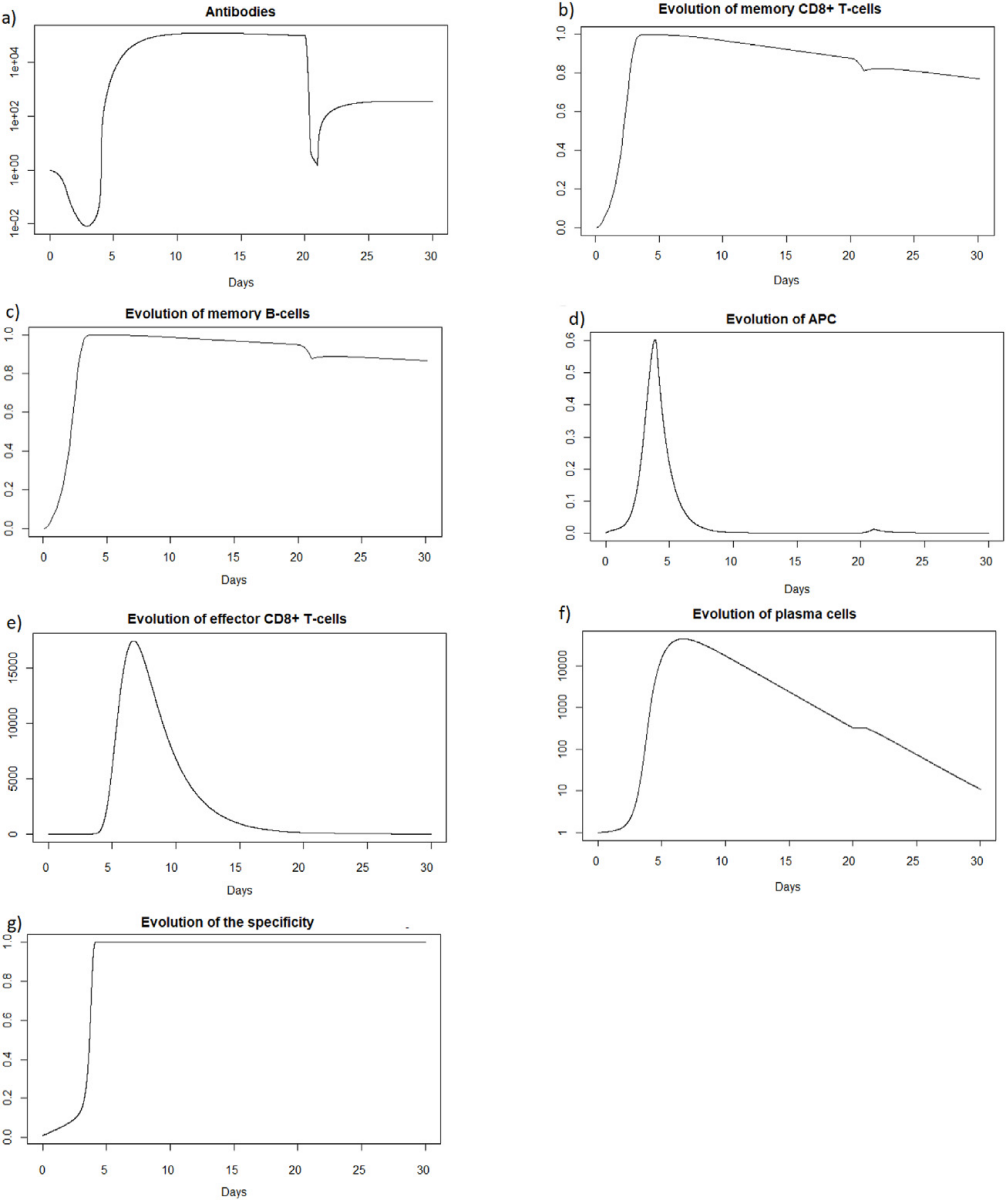
(a) Evolution of antibodies, (b) evolution of memory *CD*8^+^ T-cells, (c) evolution of memory B-cells, (d) evolution of APC, (e) evolution of effector cells, (f) evolution of plasma cells, and (g) evolution of the specificity after IAV reinfection at t = 20 days.

We then consider a new infection 20 days later with the same virus and the same intensity i.e., *V* = 0.8 to be able to compare the two immune responses. We then look at how the immune system reacts to this second infection which took place in the presence of antibodies and memory cells created during the first infection. See Fig. 2, Fig. 3.

We observe Fig. 2 that the reinfection does not invade the body. A very weak peak appears after the reinfection on the evolution of the viral load and the population of infected cells which corresponds to the encounter with the pathogen. However the immune system seems to react so quickly that the infection cannot spread. If we look at Fig. 3, we observe that before the reinfection there are antibodies in very large quantities. Thus at the time of the reinfection, we observe a very sharp decrease in antibodies which corresponds to the very fast mobilization against the pathogen. We also observed a very slight decrease in the populations of memory cells which were ready to differentiate to fight the pathogen, but the antibodies already present were sufficiently effective. In the case of a very short reinfection, antibodies seems to control the reinfection without the need for the memory B and T-cells. Antibodies already present act very quickly and are sufficient to eliminate the pathogen before it causes disease.

**Fig. 4:**
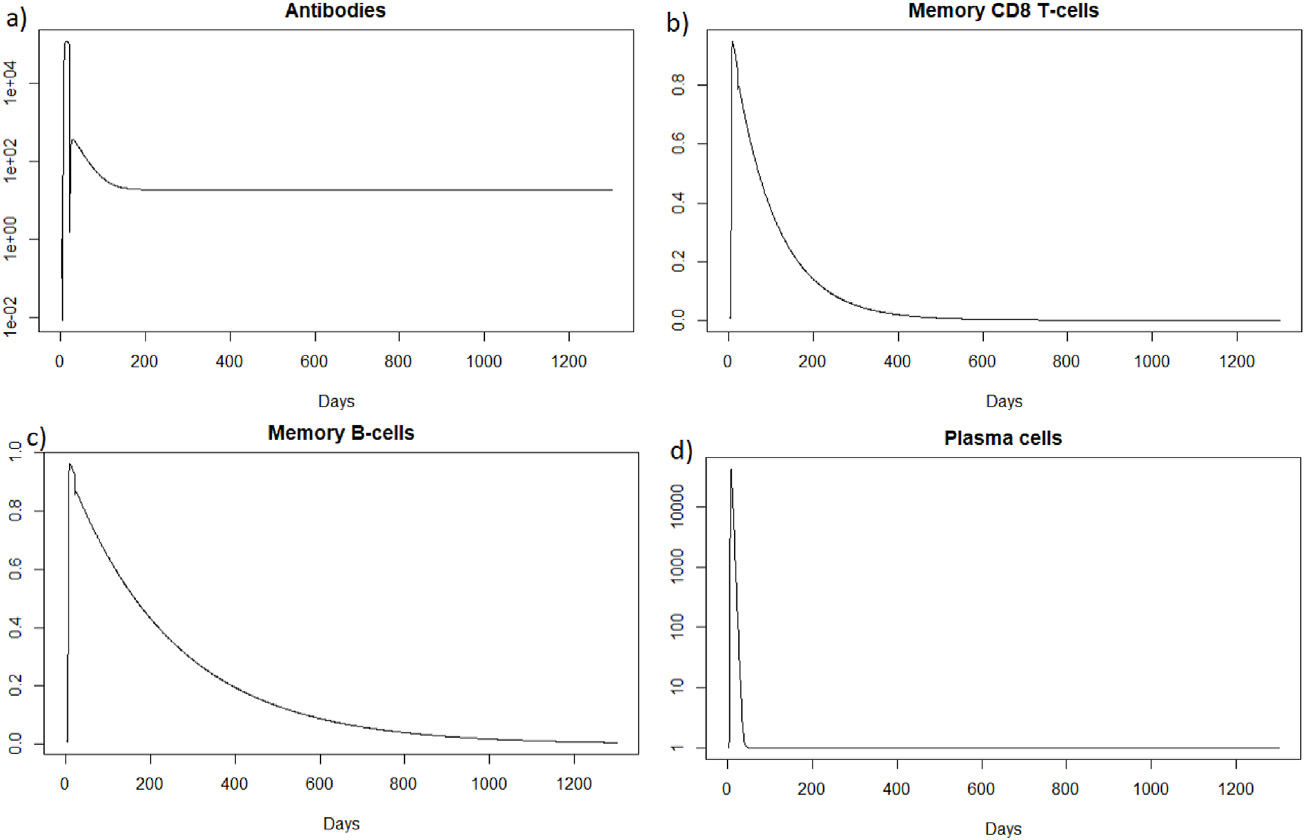
Long-term dynamics after IAV reinfection at t = 20 days of (a) antibodies, (b) memory *CD*8^+^ T-cells, (c) memory B-cells and (d) plasma cells.

We can see Fig. 4 that the steady state *#*1 is reached because *R*_0_ is less than 1 (See 3.1, 3.2). Indeed using the values from Table 2, we obtain the steady state *#*1:

(*H*, *I*, *M*, *T*_*m*_, *E*, *B*_*m*_, *P*, *V*, *A*, *S*, *D*) = (0.999, 0, 5.6 × 10^−7^, 0, 0, 0.002, 1, 0, 18.605, 1, 0.001). We can directly see in Fig. 2 that this steady state is reached for the components *H*, *I*, *D*, and *V*. We can also see Fig. 3 that *E* (e) reaches 0, *S* (g) reaches 1 and that *M* (d) is close to 0. If we plot the dynamic of the other components, for example for *t* = 1200, we can also observe that the other components reach the steady state values. Indeed, the plasma cells reache 1, and the memory cells reache 0, and the antibodies reache 18.6.

#### 3.3.2 IAV reinfection after two months

We then consider a new infection two months after the first infection, also with the same virus and the same intensity i.e., 8.10^9^ *particle*/*mL*. We then look at how the immune system reacts to this reinfection two months after.

**Fig. 5:**
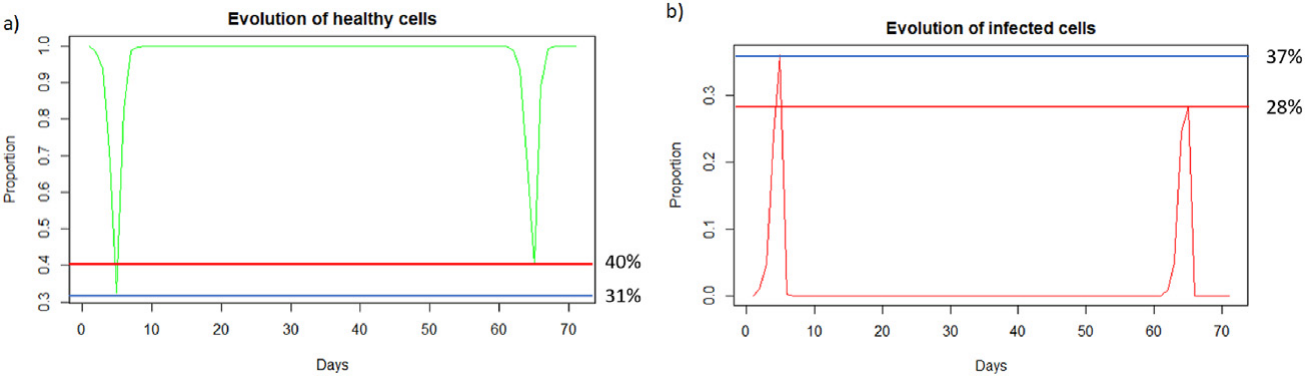
(a) Evolution of healthy cells and (b) infected cells after IAV reinfection at two months. The blue line corresponds to the percentage of cells at the first infection and the red line corresponds to the percentage of cells at the second infection.

**Fig. 6:**
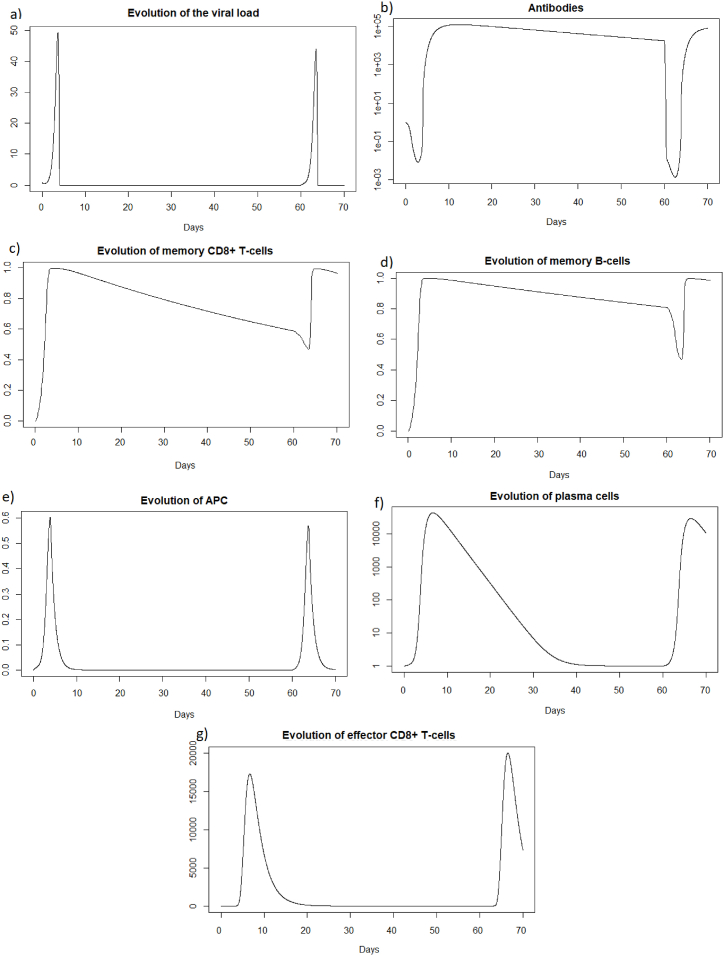
Evolution of (a) viral load, (b) antibodies, (c) memory *CD*8^+^ T-cells, (d) memory B-cells, (e) APC, (f) plasma cells, and (g) effector cells after IAV reinfection at two months.

We can observe in Fig. 5 that unlike a very rapid reinfection (see 3.3.1), in this case the individual becomes ill and all the components of the immune system will be used. We can see Fig. 5 that at the first encounter with the pathogen, the healthy cells decreased to a proportion of 31 % (blue line) while upon reinfection 2 months later the healthy cells decreased to a proportion of 40 % (red line). There are therefore 9% more healthy cells during reinfection. The proportion of infected cells, Fig. 5, amounted to 37 % (blue line) while at reinfection 2 months after the proportion of infected cells was 28 % (line red). There are therefore 9% fewer infected cells during reinfection, so the immune system seems to better control the infection when there is an immune memory. The peak of viral load, Fig. 6 (a), is less significant in the case of reinfection despite an equivalent initial dose of 8.10^9^ *particle*/*mL* which shows that reinfection is better controlled than during a first infection.

We see in Fig. 6 that after the first infection, the immune system begins to store memory CD8^+^ T-cells. They decrease slowly, and then are very quickly mobilized to respond to the second infection. In the case of reinfection after 2 months, unlike a short reinfection, memory CD8^+^ T-cells are needed to control the infection. Their population decreases during reinfection because this mobilization is stronger than the production of new memory cells. Then they grow back very quickly once the peak has passed to store even more memory cells in case a third infection occurs. Effector cells (see Fig. 6 (g)) have a higher peak during the second infection, as memory CD8^+^ T-cells allowed greater deployment of effector cells to respond to infection. There is also an increase in memory B-cells after the first infection, but this population decreases more slowly. During the second infection, they are also very quickly mobilized. The plasma cells seem to have the same peak during the first and second infection, so they do not seem to be the most important elements during the reinfection 2 months later, perhaps because there was still a very large quantity of antibodies which could be mobilized directly against the reinfection.

**Fig. 7:**
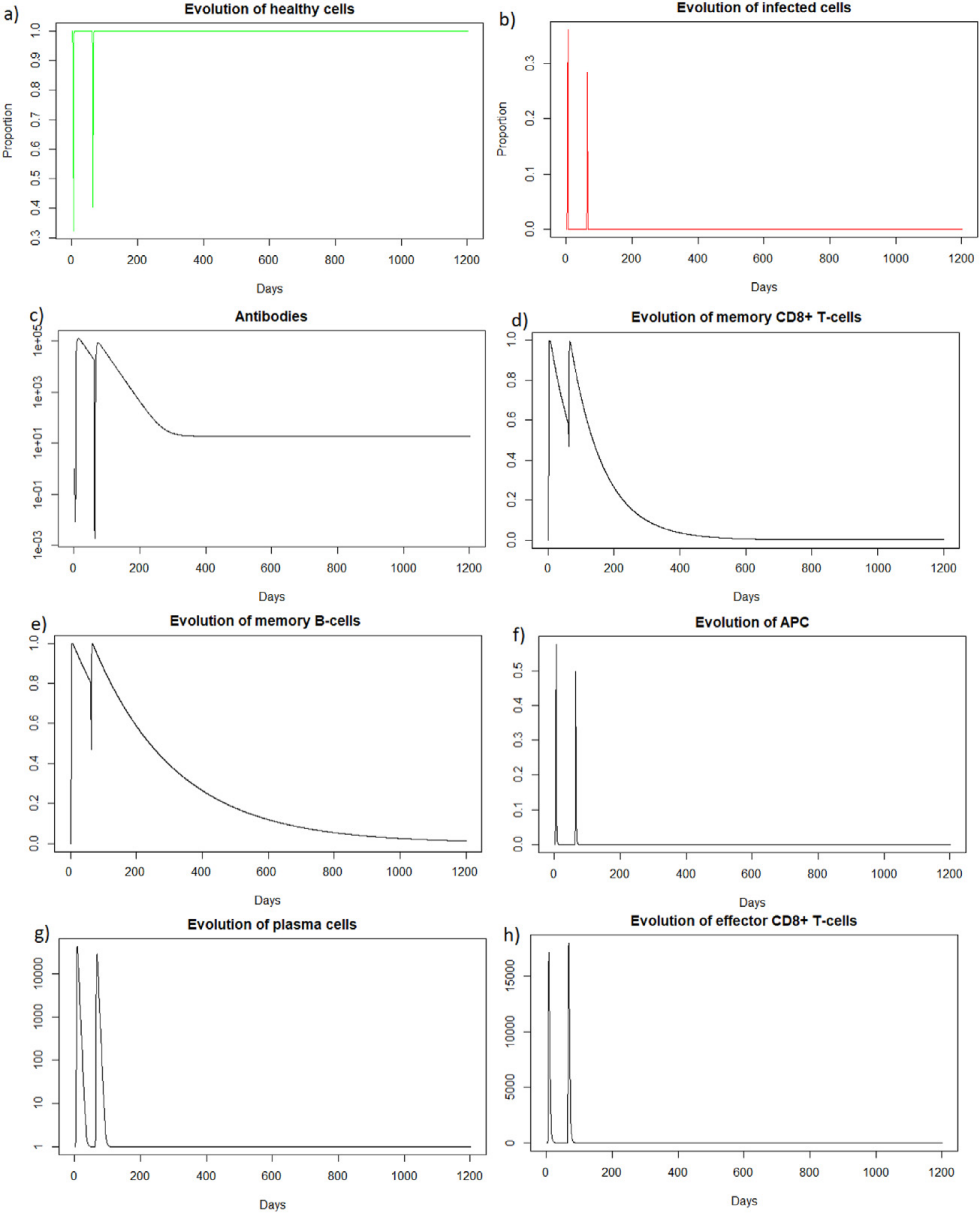
Long dynamics after IAV reinfection at two months of (a) healthy cells, (b) infected cells, (c) antibodies, (d) memory *CD*8^+^ T-cells, (e) memory B-cells, (f) APC, (g) plasma cells and (h) effector cells.

As for a reinfection with a short delay, we can see Fig. 7 that the steady state *#*1,

(*H*, *I*, *M*, *T*_*m*_, *E*, *B*_*m*_, *P*, *V*, *A*, *S*, *D*) = (0.999, 0, 5.6 × 10^−7^, 0, 0, 0.002, 1, 0, 18.605, 1, 0.001), is reached also because *R*_0_ is less than 1 (See 3.1, 3.2).

#### 3.3.3 IAV reinfection after 400 days

We are now looking a reinfection at 400 days when the immune response has greatly diminished.

**Fig. 8:**
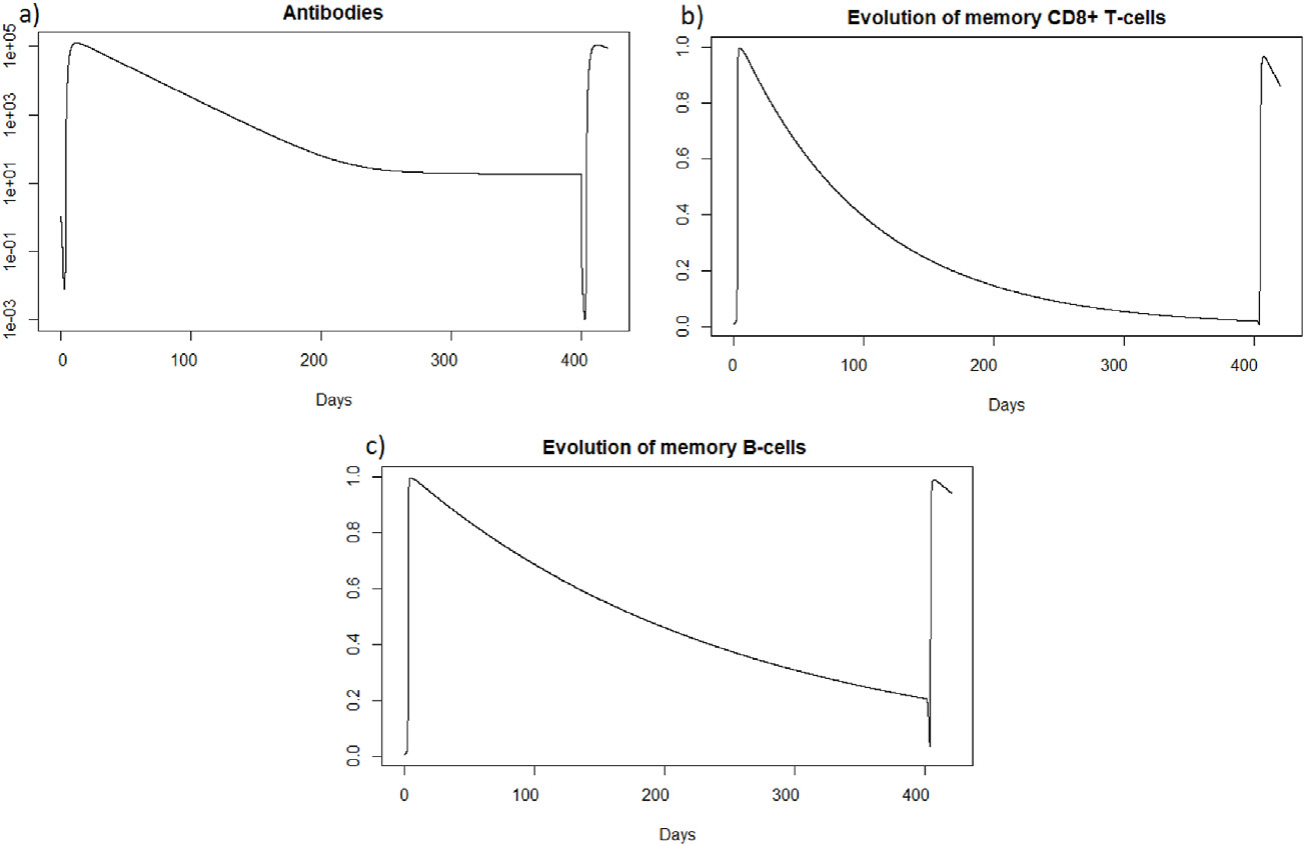
Evolution of (a) antibodies, (b) memory CD8^+^ T-cells and (c) memory B-cells after IAV reinfection at 400 days.

We see Fig. 8 that before 400 days there are almost no more memory CD8^+^ T-cells, and there are few memory B-cells and antibodies. As immune memory is very weak, the immune response will not be much more effective than in the case of a first infection.

**Fig. 9:**
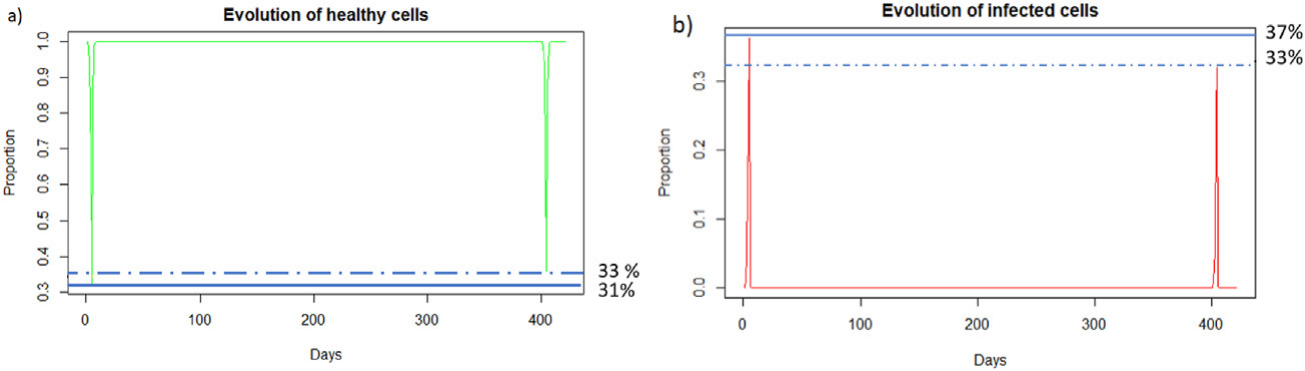
Evolution of (a) healthy and (b) infected cells after IAV reinfection at 400 days. The blue line corresponds to the percentage of cells at the first infection and the red line corresponds to the percentage of cells at the second infection.

We can see in Fig. 9 that after a reinfection at 400 days, the healthy cells decreased to a proportion of 33%. There are therefore only 2% more healthy cells compared to the first infection whereas with reinfection at 2 months (see Section 3.3.2), there was a difference of 9%. We can see Fig. 9 that the proportion of infected cells during reinfection was 33 % (red line). There are therefore only 4% fewer infected cells compared to the first infection whereas with reinfection at 2 months (see Section 3.3.2), there was a difference of 9%. The immune response for a reinfection after 400 days appears less effective than for a reinfection after two months.

In the case of Influenza A, if the second infection does not occur a few months after the first infection, the immune response will not be more effective than in the case of a first encounter with the virus.

**Fig. 10:**
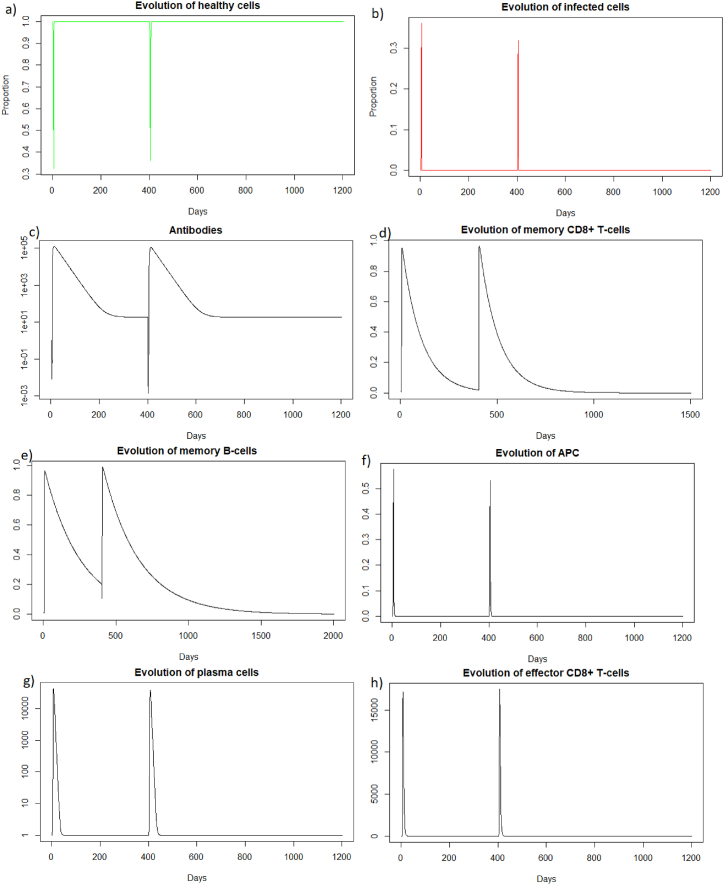
Long dynamics after IAV reinfection at 400 days of (a) healthy cells, (b) infected cells, (c) antibodies, (d) memory *CD*8^+^ T-cells, (e) memory B-cells, (f) APC, (g) plasma cells and (h) effector cells.

As for the two other cases of reinfection, we can see that the steady state *#*1,

(*H*, *I*, *M*, *T*_*m*_, *E*, *B*_*m*_, *P*, *V*, *A*, *S*, *D*) = (0.999, 0, 5.6 × 10^−7^, 0, 0, 0.002, 1, 0, 18.605, 1, 0.001), is reached again because *R*_0_ is less than 1 (See 3.1, 3.2). See the long time dynamics, Fig. 10.

In the same way as for influenza A simulations, we simulate the immune response of a host to SARS-CoV-2 reinfection.

#### 3.3.4 SARS-CoV-2 reinfection after twenty days

We then consider a new infection 20 days later with the same virus and the same intensity i.e. *V* = 0.8. We then look at how the immune system reacts to this second infection which took place in the presence of the antibodies and memory cells created during the first infection.

**Fig. 11:**
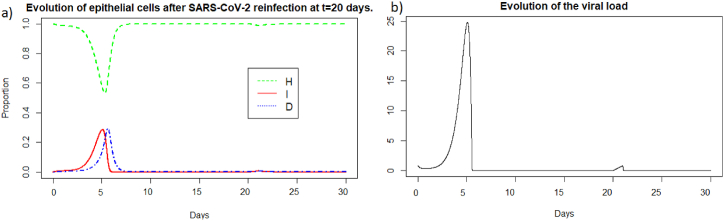
Evolution of (a) epithelial cells and (b) the viral load after SARS-COV-2 reinfection at t = 20 days.

**Fig. 12:**
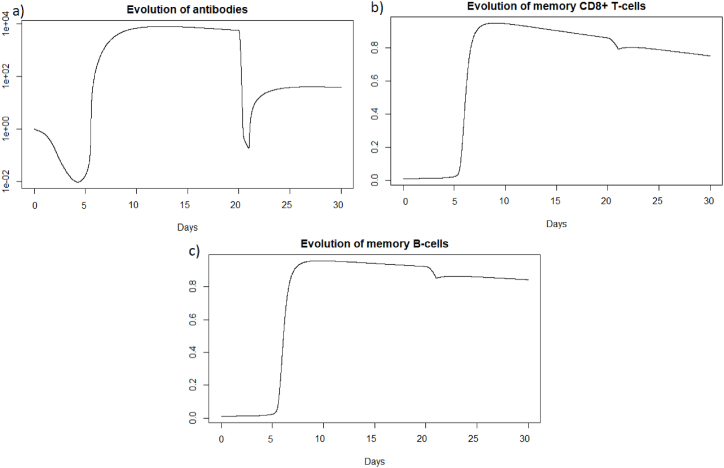
Evolution of (a) antibodies, (b) memory CD8^+^ T-cells and (c) memory B-cells after SARS-CoV-2 reinfection at t = 20 days.

We observe in Fig. 11 that the reinfection does not invade the body as in the case of influenza A. A very weak peak appears after the reinfection on the evolution of the viral load and the population of infected cells which corresponds to the encounter with the pathogen. We can observe that with the data used for SARS-CoV-2, the peak during the first infection is lower than for Influenza A and the proportion of healthy cells remains higher. However as for Influenza A (see Section 3.3.1), the immune system seems to react so quickly that the infection cannot spread. If we look at Fig. 12, we observe that we have the same dynamics as for influenza A.

**Fig. 13:**
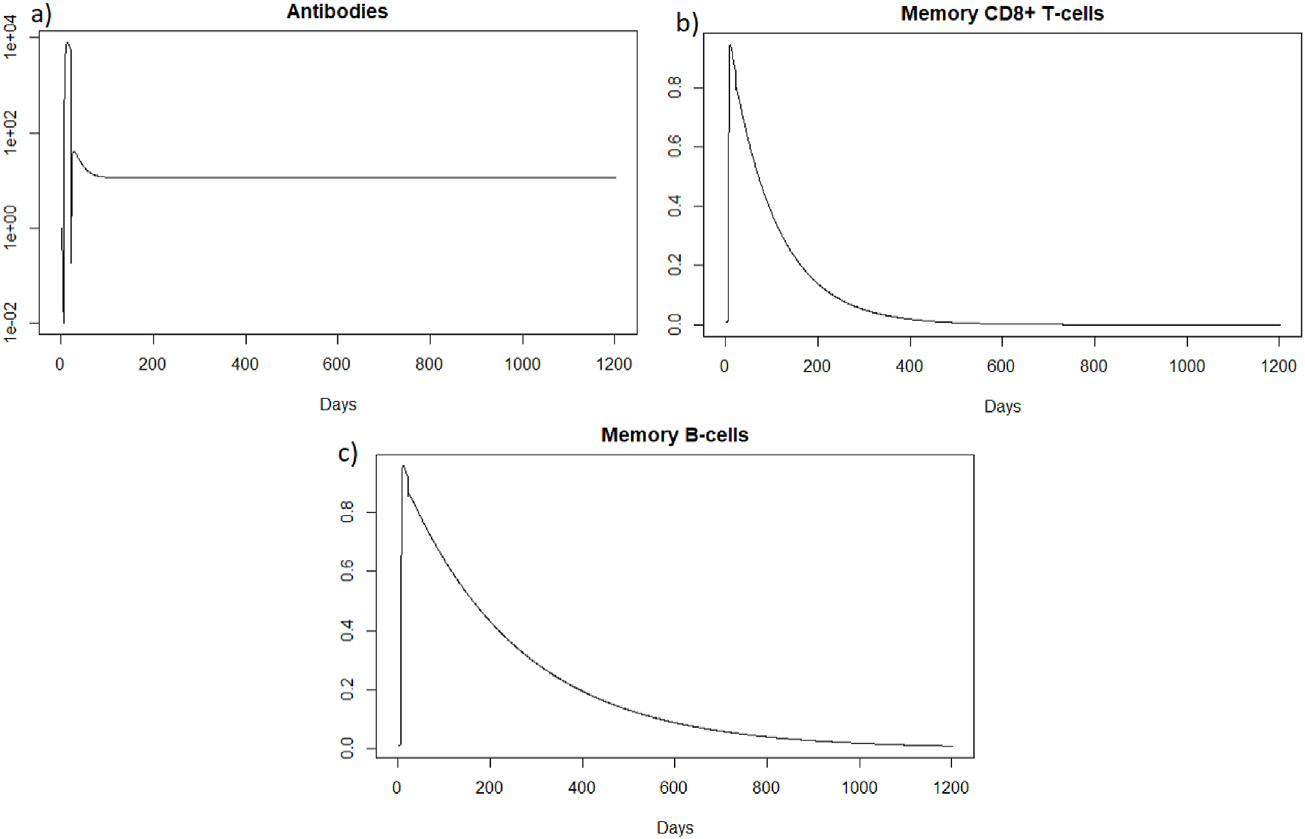
Long-term dynamics of (a) antibodies, (b) memory *CD*8^+^ T-cells and (c) memory B-cells after SARS-CoV-2 reinfection at t = 20 days.

We can see that the steady state *#*1 is reached because *R*_0_ is less than 1 (See 3.1, 3.2). Using the values from Table 2, we obtain the steady state *#*1:

(*H*, *I*, *M*, *T*_*m*_, *E*, *B*_*m*_, *P*, *V*, *A*, *S*, *D*) = (0.999, 0, 5.6 × 10^−7^, 0, 0, 0.002, 1, 0, 11.429, 1, 0.001). We can directly see in Fig. 11 that this steady state is reached for the components *H*, *I*, *D*, and *V*. If we plot the dynamic of some other components, see Fig. 13, for *t* = 1200, we can also observe that the other components approach the steady state values. Indeed, the memory cells reach 0, and antibodies reach 11.42.

#### 3.3.5 SARS-CoV-2 reinfection after two months

We then consider a new infection two months after the first infection, also with the same virus and the same intensity i.e. 8.10^9^ *particle*/*mL*. We then look at how the immune system reacts to this reinfection two months after.

**Fig. 14:**
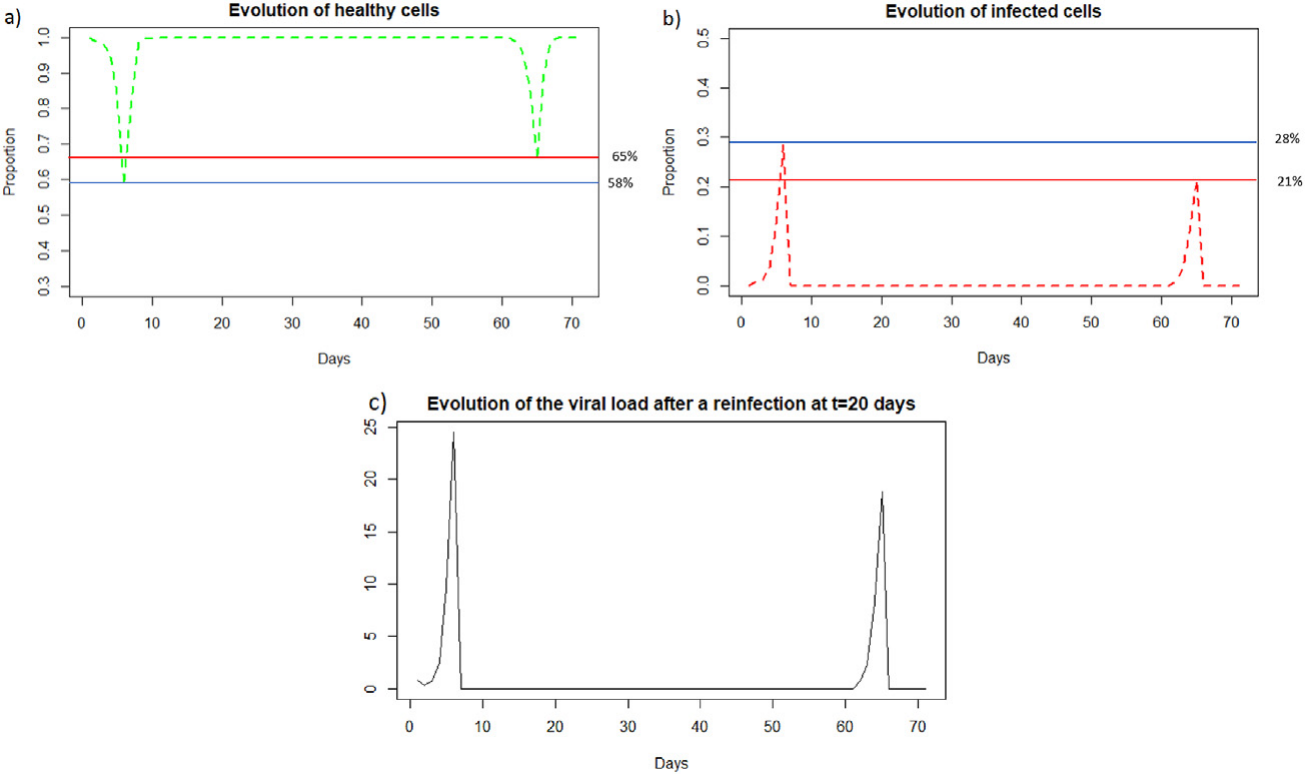
Evolution of (a) healthy cells, (b) infected cells and (c) viral load after SARS-CoV-2 reinfection at two months. The blue line corresponds to the percentage of cells at the first infection and the red line corresponds to the percentage of cells at the second infection.

**Fig. 15:**
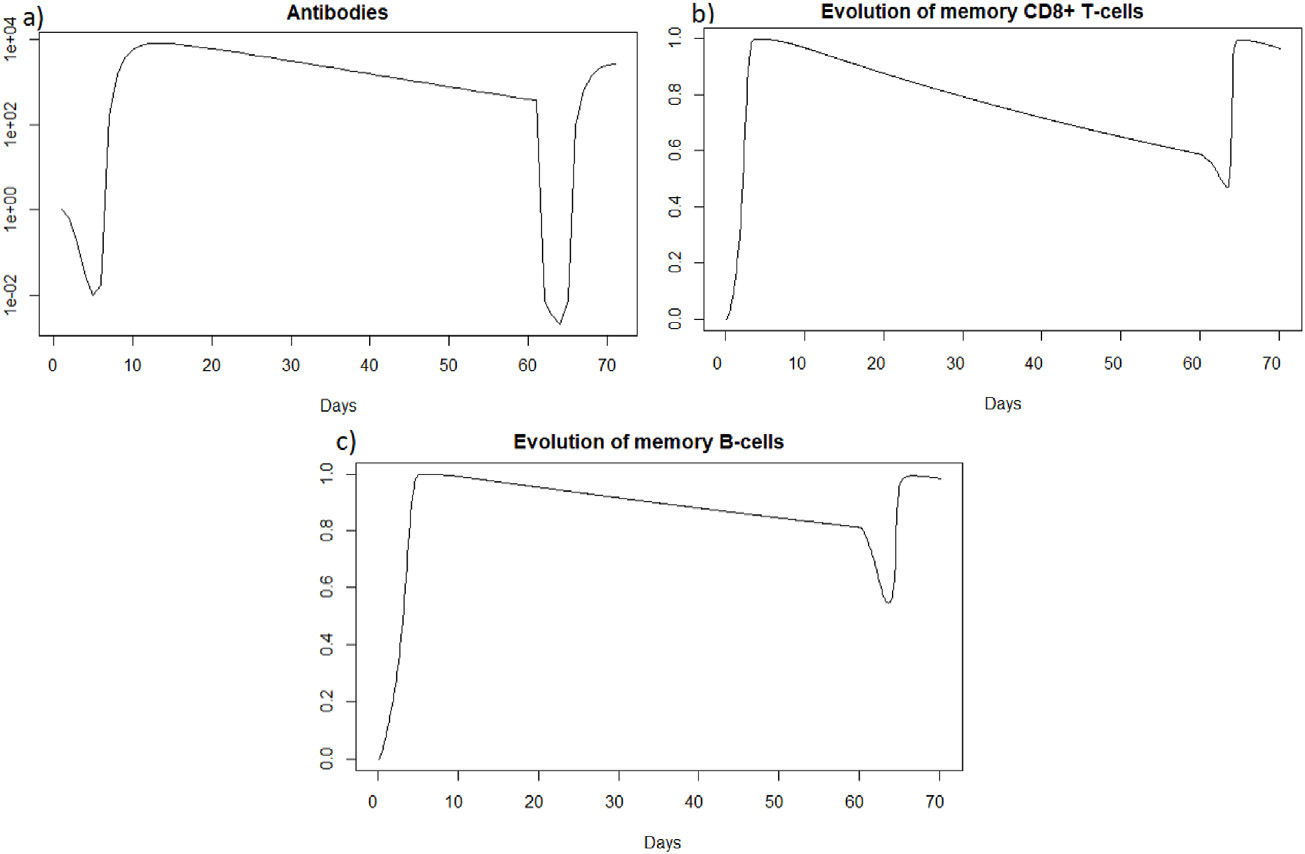
Evolution of (a) antibodies, (b) memory CD8^+^ T-cells and (c) memory B-cells, after SARS-CoV-2 reinfection at two months.

We can observe in Fig. 14 that unlike a very rapid reinfection (see Section 3.3.4), in this case the individual becomes ill and all the components of the immune system will be use, see Fig. 15, as for the reinfection by IAV. We can see Fig. 14 (a) that at the first encounter with the pathogen, the healthy cells decreased to a proportion of 58 % (blue line) while upon reinfection 2 months later the healthy cells decreased to a proportion of 65 % (red line). There are therefore 7% more healthy cells during reinfection. The proportion of infected cells, Fig. 14 (b), amounted to 28 % (blue line) while at reinfection 2 months after the proportion of infected cells was 21 % (red line). There are therefore 7% fewer infected cells during reinfection, so the immune system seems to better control the infection when there is an immune memory. The peak of viral load, Fig. 14 (c), is less significant in the case of reinfection despite an equivalent initial dose of 8.10^9^ *particle*/*mL* which shows that reinfection is better controlled than during a first infection.

**Fig. 16:**
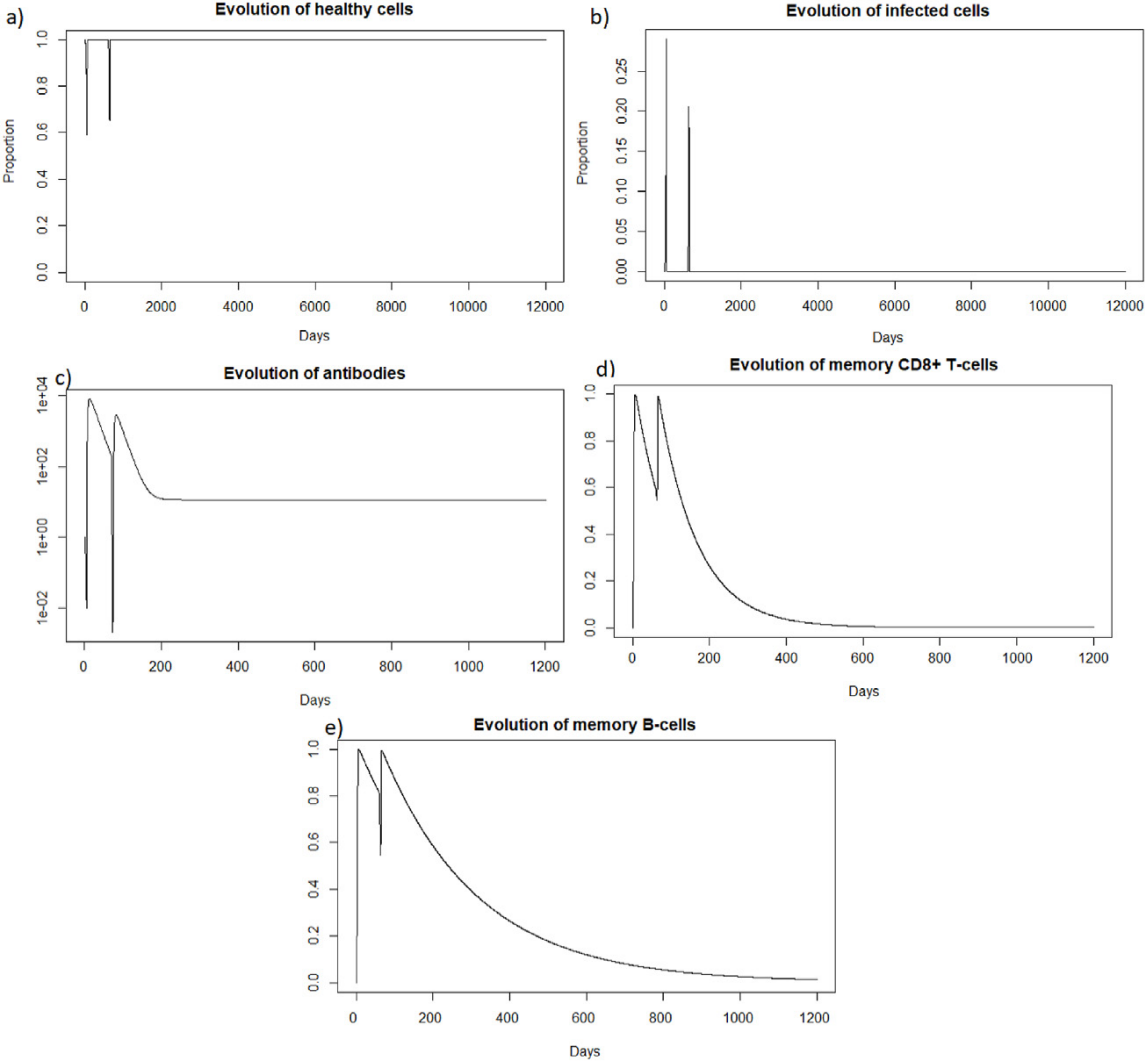
Long dynamics of (a) healthy cells, (b) infected cells, (c) antibodies, (d) memory *CD*8^+^ T-cells, and (e) memory B-cells after SARS-CoV-2 reinfection at two months.

As for a reinfection with a short delay, we can see Fig. 16 that the steady state *#*1,

(*H*, *I*, *M*, *T*_*m*_, *E*, *B*_*m*_, *P*, *V*, *A*, *S*, *D*) = (0.999, 0, 5.6 × 10^−7^, 0, 0, 0.002, 1, 0, 11.429, 1, 0.001), is reached also because *R*_0_ is less than 1 (see 3.1, 3.2).

#### 3.3.6 SARS-CoV-2 reinfection after 400 days

**Fig. 17:**
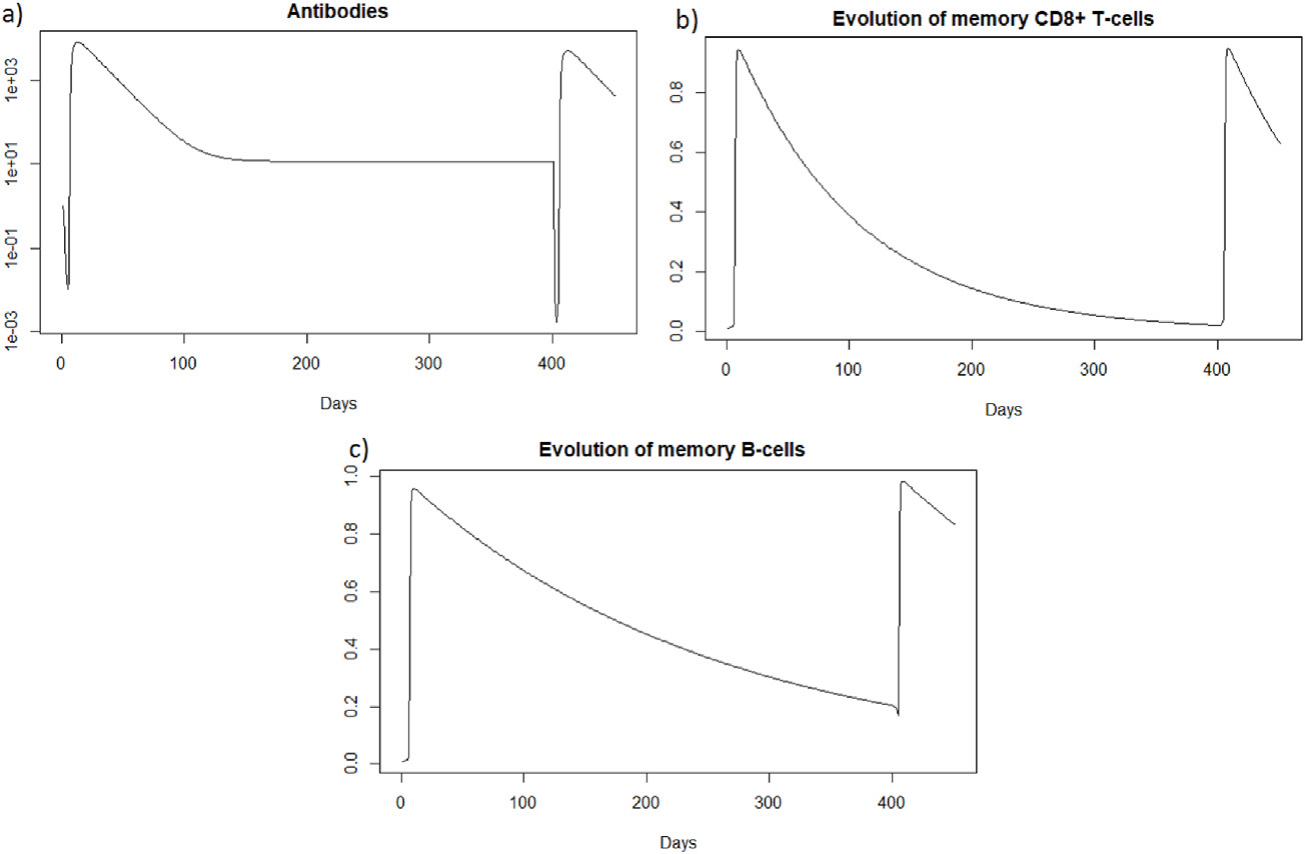
Evolution of (a) antibodies, (b) memory CD8^+^ T-cells and (c) memory B-cells after SARS-CoV-2 reinfection at 400 days.

We are now investigating the reinfection at 400 days, when the immune response has greatly diminished. We see that before 400 days there are almost no more memory CD8^+^ T-cells, and there are few memory B-cells and antibodies; see Fig. 17.

**Fig. 18:**
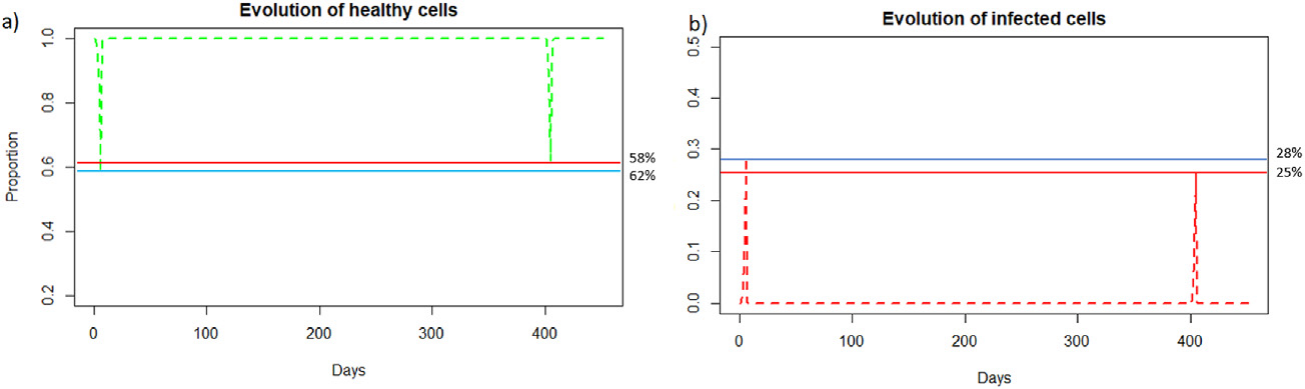
Evolution of (a) healthy cells and (b) infected cells after SARS-CoV-2 reinfection at 400 days.

We can see in Fig. 18 (a) that after a reinfection at 400 days, the healthy cells decreased to a proportion of 62%. There are therefore only 4% more healthy cells compared to the first infection whereas with reinfection at 2 months, see 3.3.2, there was a difference of 7%. We can see Fig. 18 (b), that the proportion of infected cells was 25 % in the second infection (red line). There are therefore only 3% fewer infected cells compared to the first infection whereas with reinfection at 2 months, see 3.3.2, there was a difference of 7%. In the case of SARS-CoV-2, the memory immune response appears stronger when it is close to the first infection, as for influenza A. As with influenza A, in the case of late reinfections with SARS-Cov-2, the immune response looks like the primary immune response because immune memory is weak. Indeed, there is no great difference in the reduction in the number of cells that become infected or remain healthy compared with the first response. As in the case of a primary infection, new memory B-cells, memory T-cells and antibodies are created to respond effectively to a third infection if it occurs rapidly.

**Fig. 19:**
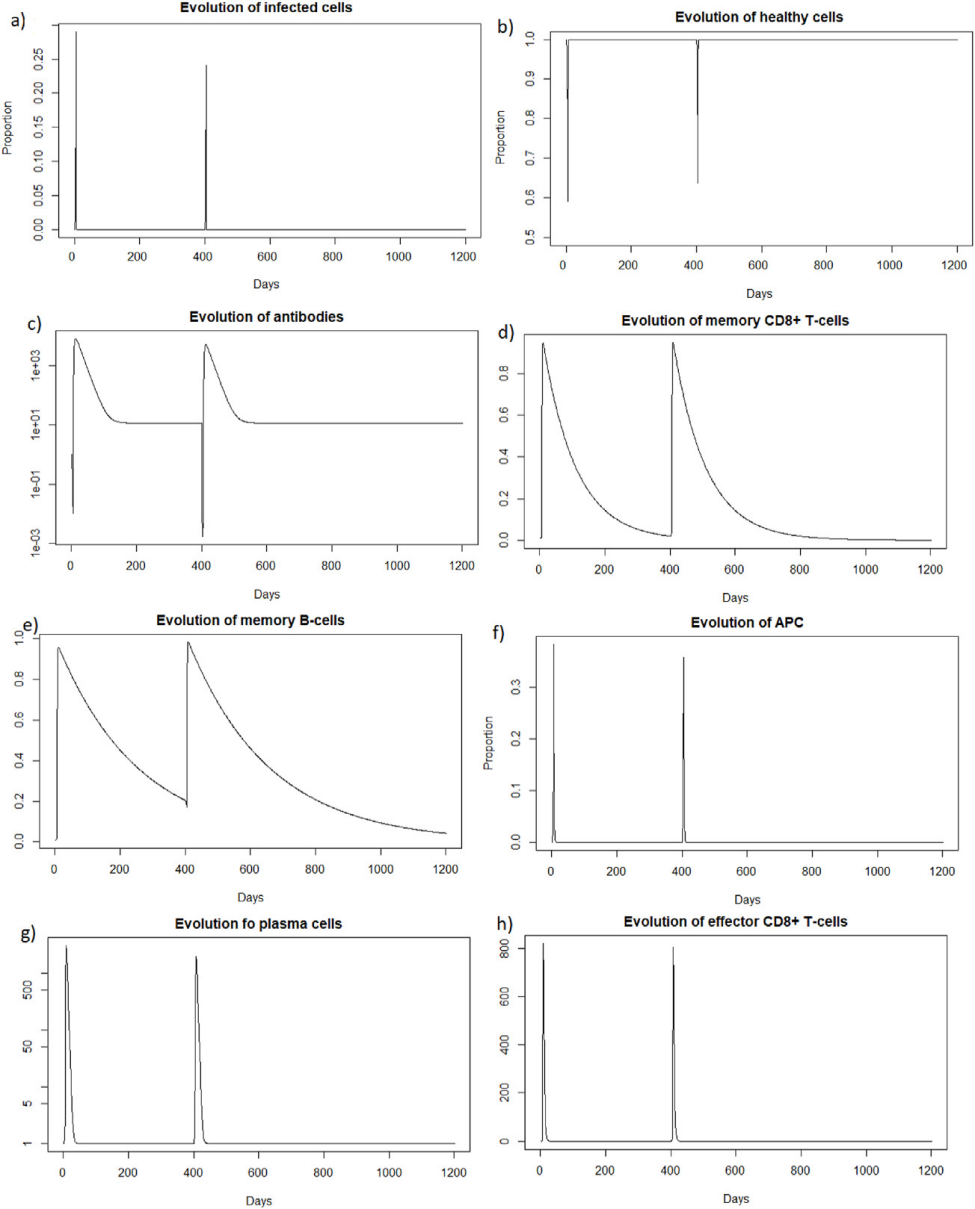
Long dynamics of (a) infected cells, (b) healthy cells, (c) antibodies, (d) memory *CD*8^+^ T-cells, (e) memory B-cells, (f) APC, (g) plasma cells and (h) effector cells after SARS-CoV-2 reinfection at 400 days.

As for the two other cases of reinfection, Fig. 19, we can see that the steady state *#*1,

(*H*, *I*, *M*, *T*_*m*_, *E*, *B*_*m*_, *P*, *V*, *A*, *S*, *D*) = (0.999, 0, 5.6 × 10^−7^, 0, 0, 0.002, 1, 0, 11.429, 1, 0.001), is reached also because *R*_0_ is less than 1 (See subsection 3.1 and 5).

#### 3.3.7 Impact of the viral load received during reinfection

**Fig. 20:**
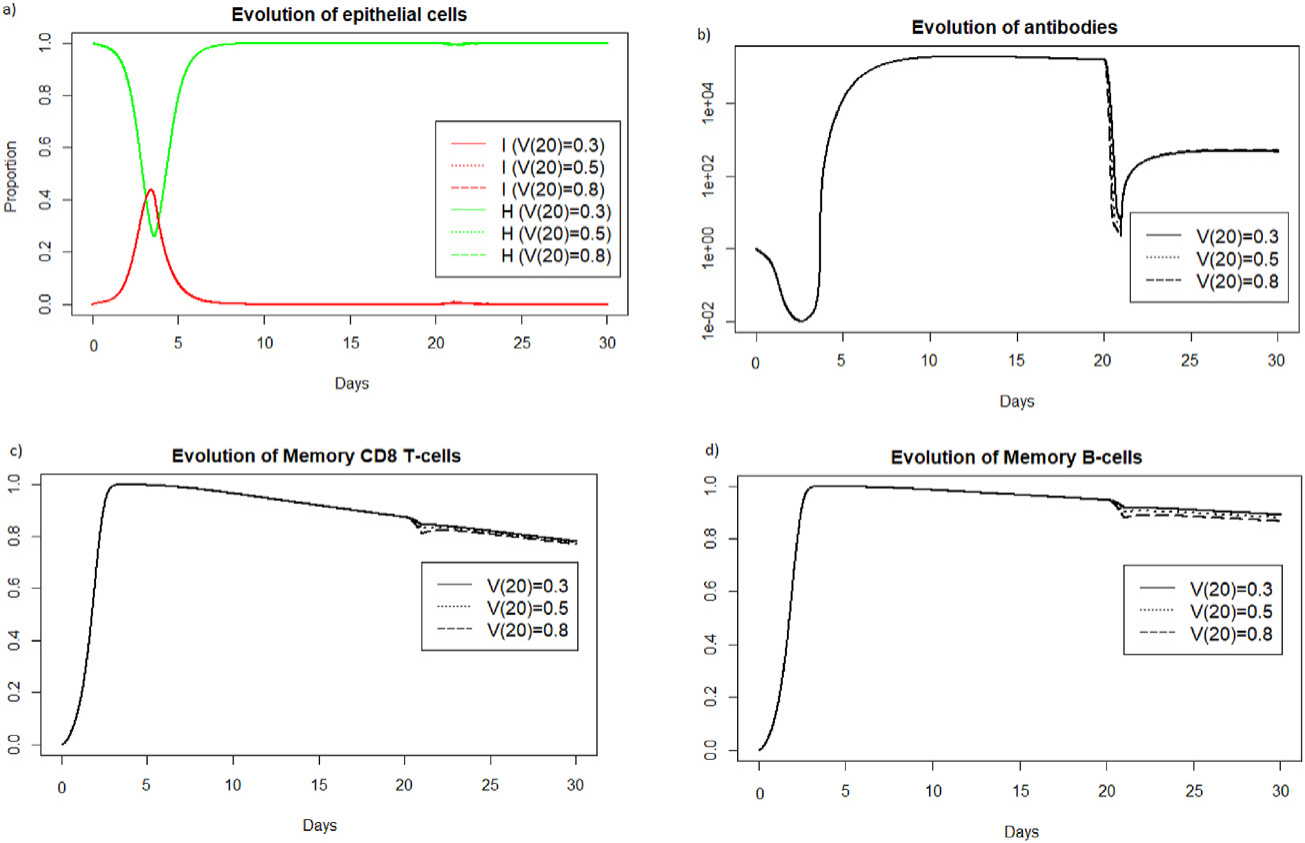
Evolution of a) epithelial cells, b) antibodies, c) memory T-cells and d) memory B-cells with respect to the viral load received during reinfection at 20 days.

In 3.3.1, 3.3.2, 3.3.3 we focused on IAV reinfections with the same intensity as during the first infection. We study now the impact of varying the viral load received during IAV reinfection. In Fig. 20, we consider different viral loads intensities received during reinfection at 20 days after the first encounter with the pathogen.

We can see in Fig. 20 a) that for the three different values of V (20), the reinfection does not invade the body. If we look at b), we see that the immune system mobilizes fewer antibodies to prevent the spread of infection when the viral load is lower. We also observe in Fig. 20 c) and d) that the lowest viral load curve is above the other curves over the period of 20–30 days. This means that the higher the viral load, the more memory cells are mobilized.

**Fig. 21:**
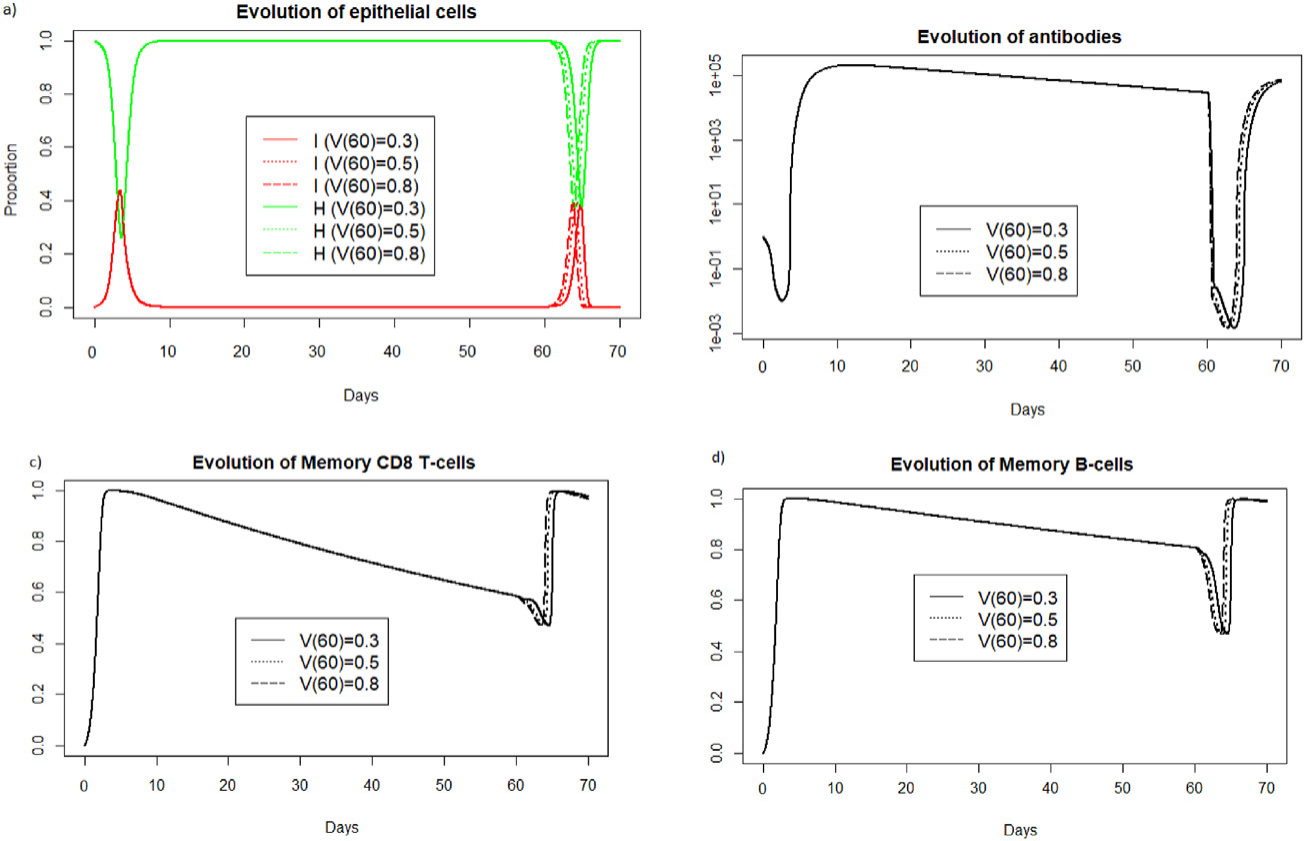
Evolution of a) epithelial cells, b) antibodies, c) memory T-cells and d) memory B-cells with respect to the viral load received during reinfection at two months.

In Fig. 21, we consider different viral loads intensities received during reinfection at two months.

We can see Fig. 21 that a lower viral load during reinfection is associated with a later response. The mobilization of antibodies and memory cells is shifted over time depending on the viral load received during reinfection. The more intense the viral infection, the more rapidly the immune system responds.

**Fig. 22:**
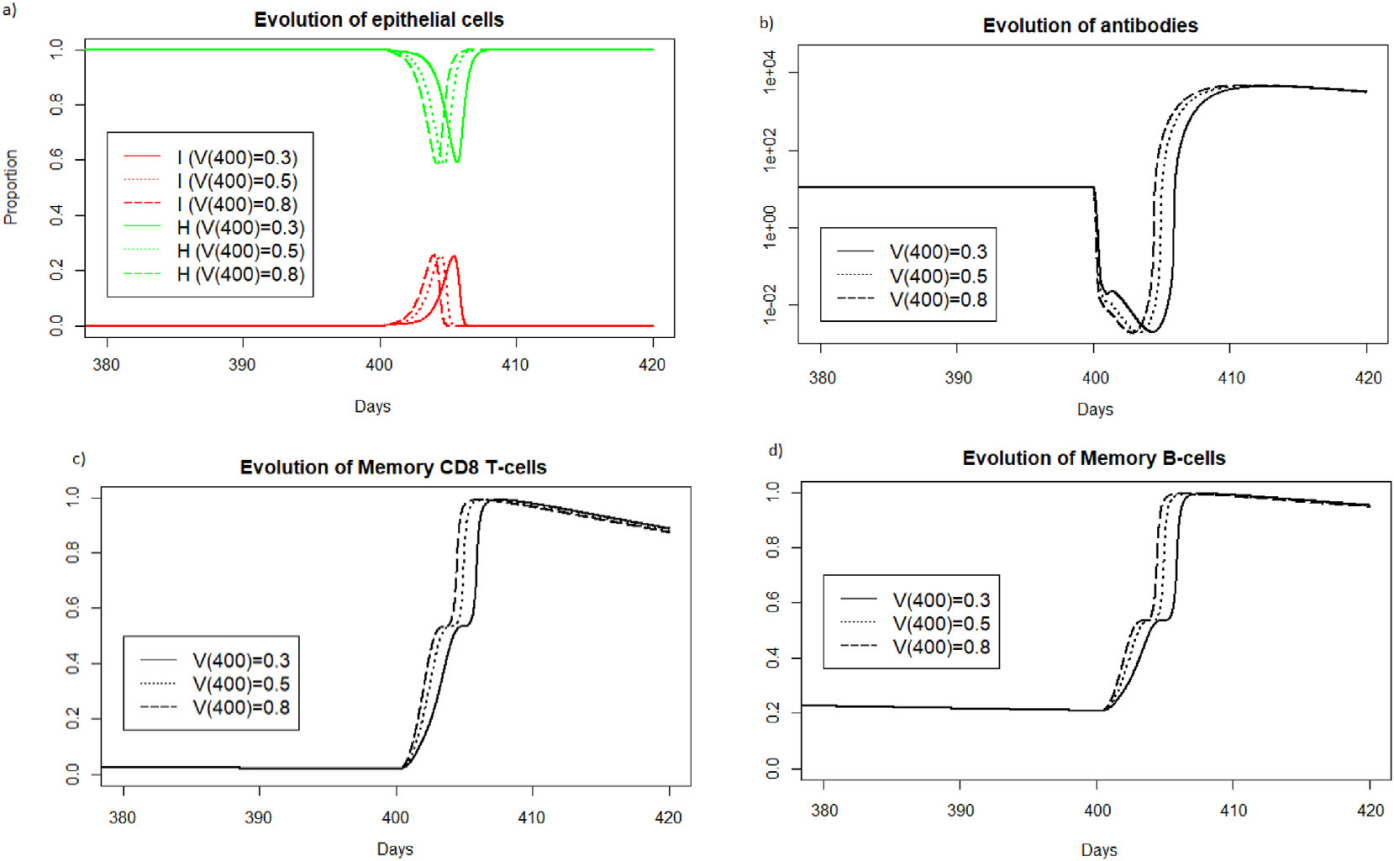
Evolution of a) epithelial cells, b) antibodies, c) memory T-cells and d) memory B-cells with respect to the viral load received during reinfection at 400 days after the first encounter with the pathogen.

In Fig. 22, we consider different viral loads intensities received during reinfection at 400 days. We display the simulations only for the period surrounding the reinfection to better observe what is happening on the graphs.

We can see Fig. 22, as in the case of reinfection 2 months later, that the response time of the memory immune system is shifted in time depending on the intensity of the viral load received during reinfection.

## 4 Discussion *&* conclusion

In this paper we investigated the immune responses against IAV and SARS-CoV-2 secondary infections, in the presence of immune memory that formed after primary infections with these viruses. To this end, we adapted a previously-published in-host mathematical model (Sadria & Layton, 2021) to include also the dynamics of memory responses.

To understand the role of immune memory on the dynamics of this model, we first focused on the long-term behaviour and we identified all virus-free steady states and their stability. Two steady states seemed of interest regarding the immune response. A healthy virus-free steady state with memory cells always stable when *R*_0_ < 1 which ensures the permanent elimination of the virus and the formation of a healthy, persistent antiviral immune response under this condition. We also obtain conditions concerning a steady state to be unstable where immune memory is present but the individual is close to death, it's interesting to know the conditions for not remaining in this state.

After looking at different reinfections with different delays, we can conclude that, in the case of a reinfection with a very short delay (20 days for instance), the most important component of the immune memory response seems to be represented by the antibodies. Indeed, they appear to control reinfection without the need for memory B and CD8^+^ T-cells. Antibodies already present in the body act very quickly and are sufficient to eliminate the pathogen before it causes disease. For reinfection with IAV two months later, the memory immune response reduced the infected cells by 9% and increased the healthy cells by 9% compared to the first infection. For SARS-CoV-2 infection, the memory immune response reduced infected cells by 7% and increased healthy cells by 7%. In this case, memory cells are needed in addition to the antibodies already present in the body, to control the infection. For reinfection 400 days later, when immune memory is low, the immune response reduced infected cells by 4% and increased healthy cells by 2% compared with the first infection in the case IAV infection. For SARS-CoV-2 infection, infected cells were reduced by 3% and healthy cells increased by 4%. There was a slight improvement in the second response, thanks to the antibodies and memory B-cells (since there were no longer any memory CD8^+^ T-cells). In the case of reinfection at 400 days, there is still a slight improvement in immune response thanks to immune memory. However, this improvement is much less significant than in the case of intermediate reinfection. Indeed, in the case of late reinfection, the immune response looks like the primary immune response. There is not a big difference in the reduction in the number of cells that become infected or that remain healthy compared to the first response. As in the case of a primary infection, there is a creation of new memory B-cells, memory T-cells and antibodies to respond effectively to a third infection if it occurs quickly. Finally, the immune response after a secondary infection is more efficient when the reinfection occurs at a shorter time, i.e., when there is a high immune memory. The closer the reinfection is to the first encounter with the pathogen, the more the immune memory protects the host against the infection.We also varied the viral load received during reinfection. We can conclude that in the case of a reinfection with a very short delay (20 days for instance), the higher the viral load, the greater the number of antibodies and memory cells mobilized to eliminate the pathogen. In the case of later reinfection, a higher viral load is associated with a more rapid immune system response and a more rapid impact on the epithelial cells.

This study is only a first step in the investigation of a complex in-host dynamics observed during the re-infection with different viruses. There are many ways in which this work can be continued, and this will be the subject of future studies. For example, a more in-depth analytical investigation of the global stability of the steady states identified here could help shed light on the importance of initial conditions on the persistence of these steady states.

## CRediT authorship contribution statement

**Mathilde Massard:** Writing – original draft, Software, Methodology, Conceptualization. **Bruno Saussereau:** Writing – review & editing, Validation, Supervision. **Catherine Chirouze:** Writing – review & editing, Validation. **Quentin Lepiller:** Writing – review & editing, Validation, Supervision. **Raluca Eftimie:** Writing – review & editing, Validation, Supervision. **Antoine Perasso:** Writing – review & editing, Validation, Supervision.

## Declaration of competing interest

The author is an Editorial Board Member/Editor-in-Chief/Associate Editor/Guest Editor for *Infectious Disease Modelling* and was not involved in the editorial review or the decision to publish this article.

## Appendix A Rescaling of parameters in Table 2

The values taken in (Bocharov & Romanyukha, 1994) are noted *name*^*B*^. The values taken in (Smith & Perelson, 2011) are noted *name*^*S*^.

Using the conversion 10^10^*particles* = 10^4^*TCID*_50_ given in (Bocharov & Romanyukha, 1994) and 1 mol.L^−1^ = 6∗10^20^ *particle*.*mL*^−1^ we obtain:

$$\begin{array}{cc} \gamma_{HV}^{S} & =3.2\times 10^{-5}\;\;TCID_{50}^{-1}mLday^{-1}\\ \gamma_{HV}^{S} & =3.2\times 10^{-11}\;\;particles^{-1}mLday^{-1}\\ \gamma_{HV}^{S} & =19.2\times 10^{9}\;\;M^{-1}day^{-1} \end{array}$$

Thus we have

$$\begin{array}{ll} \gamma_{HV} & =\underset{M^{-1}day^{-1}}{\underbrace{\gamma_{HV}^{S}}}\times \underset{M}{\underbrace{H^{*}}}\\ & =19.2\times 10^{9}\times 1.7\times 10^{-12}=0.033\;\;day^{-1}\\ \gamma_{VA} & =\underset{M^{-1}day^{-1}}{\underbrace{\gamma_{VA}^{B}}}\times \underset{M}{\underbrace{H^{*}}}\\ & =8.6\times 10^{12}\times 1.7\times 10^{-11}=146.2\;\;day^{-1}\\ \gamma_{VH} & =\underset{M^{-1}day^{-1}}{\underbrace{\gamma_{VH}^{B}}}\times \underset{M}{\underbrace{H^{*}}}\\ & =10^{8}\times 1.7\times 10^{-11}=1.7\times 10^{-3}\;\;day^{-1}\\ b_{IE} & =\underset{M^{-1}day^{-1}}{\underbrace{b_{IE}^{B}}}\times \underset{M}{\underbrace{H^{*}}}\\ & =10^{8}\times 1.7\times 10^{-11}=1.7\times 10^{-3}\;\;day^{-1}\\ b_{EM} & =\underset{M^{-2}day^{-1}}{\underbrace{b_{EM}^{B}}}\times \underset{M}{\underbrace{E^{*}}}\underset{M}{\underbrace{M^{*}}}\\ & =10^{32}\times 10^{-16}\times 10^{-15}=10\;\;day^{-1}\\ \gamma_{AV} & =\underset{M^{-1}day^{-1}}{\underbrace{\gamma_{AV}^{B}}}\times \underset{M}{\underbrace{H^{*}}}\\ & =8.6\times 10^{12}\times 1.7\times 10^{-11}=146.2\;\;day^{-1}\\ b_{EI} & =\underset{M^{-1}day^{-1}}{\underbrace{b_{EI}^{B}}}\times \underset{M}{\underbrace{E^{*}}}\\ & =\times 10^{8}\times 10^{-16}=10^{-8}\;\;day^{-1}\\ b_{MV} & =\underset{M^{-1}day^{-1}}{\underbrace{b_{MV}^{B}}}\times \underset{M}{\underbrace{M^{*}}}\\ & =4\times 10^{13}\times 10^{-15}=0.04\;\;day^{-1}\\ b_{PM} & =\underset{M^{-2}day^{-1}}{\underbrace{b_{PM}^{B}}}\times \underset{M}{\underbrace{M^{*}}}\underset{M}{\underbrace{M^{*}}}\\ & =10^{31}\times 10^{-15}\times 10^{-15}=10\;\;day^{-1} \end{array}$$

### Appendix B Calculation of *R*_0_

$$\left(\underset{infected}{\underbrace{V,I,}}\underset{\text{non infected}}{\underbrace{H,M,T_{m},E,B_{m},P,A,S}}\right)$$

$F=\left(\gamma_{V}I,\gamma_{HV}VH,0,0,0,0,0,0,0,0\right)$

V^-=γVASAV+γVHVH+αVV,bIEEI+aII,γHVVH+aHH,

*b*_*MD*_*H* + *b*_*MD*_*I* + *Mb*_*MD*_ + *b*_*MV*_*VM* + *a*_*M*_*M*, *b*_*TM*_*MT*_*m*_ + *γ*_*VT*_*T*_*m*_*V* + *a*_*T*_*T*_*m*_, *b*_*EI*_*IE* + *a*_*E*_*E*,

$\left(b_{BM}MB_{m}+\gamma_{VB}B_{m}V+a_{B}B_{m},a_{P}P,\gamma_{AV}SAV+a_{A}A,rPS\right)$

V^+=0,0,bHDH,bMD+bMVV+bMDHM+bMDIM,bTMM,bEMME+γVTTmV,

$\left(b_{BM}M,b_{PM}MP+\gamma_{VB}B_{m}V+a_{P}P,b_{A}P,rP\right)$

V=V^-−V^+=γVASAV+γVHVH+αVV,bIEEI+aII,γHVVH+aHH−bHDH,bMDH+bMDI+MbMD+bMVVM+aMM−(bMD+bMVV+bMDHM+bMDIM),bTMMTm+γVTTmV+aTTm−bTMM,bEIIE+aEE−(bEMME+γVTTmV),

$\left(b_{BM}MB_{m}+\gamma_{VB}B_{m}V+a_{B}B_{m}-b_{BM}M,a_{P}P-\left(b_{PM}MP+\gamma_{VB}B_{m}V+a_{P}P\right),\gamma_{AV}SAV+a_{A}A-b_{A}P,rPS-rP\right)$

$F≔\left(\begin{array}{cc} 0 & \gamma_{V}\\ \gamma_{VH}H^{*} & 0 \end{array}\right)\;\;\;\;\;\;V≔\left(\begin{array}{cc} \gamma_{VA}S^{*}A^{*}+\gamma_{VH}H^{*}+\alpha_{V} & 0\\ 0 & b_{IE}E^{*}+a_{I} \end{array}\right)$

We obtain the next generation matrix *M* below:

$$M≔FV^{-1}=\left(\begin{array}{cc} 0 & \frac{\gamma_{V}}{b_{IE}E^{*}+a_{I}}\\ \frac{\gamma_{HV}H^{*}}{\gamma_{VA}S^{*}A^{*}+\gamma_{VH}H^{*}+\alpha_{V}} & 0 \end{array}\right),$$

Thus

$$R_{0}={\left(\frac{\gamma_{HV}H^{*}\gamma_{V}}{A^{*}E^{*}S^{*}b_{IE}\gamma_{VA}+A^{*}S^{*}a_{I}\gamma_{VA}+E^{*}H^{*}b_{IE}\gamma_{VH}+E^{*}\alpha_{V}b_{IE}+H^{*}a_{I}\gamma_{VH}+a_{I}\alpha_{V}}\right)}^{1/2}.$$

## References

[bib1] Agha A.D.A., Elaiw A.M. (2022). Global dynamics of SARS-CoV-2/malaria model with antibody immune response. Mathematical Biosciences and Engineering: MBE.

[bib2] Ahmed M.E., Agha A.D Al (2023). Analysis of the in-host dynamics of tuberculosis and SARS-CoV-2 coinfection. Mathematics.

[bib3] Ahmed M.E., Alsulami R.S., Hobiny A.D. (2022). Modeling and stability analysis of within-host IAV/SARS-CoV-2 coinfection with antibody immunity. Mathematics.

[bib4] Ahmed R. Kalia V, Sarkar S. CD8 T-cell memory differentiation during acute and chronic viral infections. Madame Curie Bioscience Database *[Internet]*. 10.1007/978-1-4419-6451-9_7 20795542

[bib5] Amoddeo A. (2023). A mathematical model and numerical simulation for sars-cov-2 dynamics. Scientific Reports.

[bib6] Anaya J.-M., Shoenfeld Y., Rojas-Villarraga A., Levy R.A., Cervera R. (2013).

[bib7] Atifa A., Khan M.A., Iskakova K., Al-Duais F.S., Ahmad I. (2022). Mathematical modeling and analysis of the sars-cov-2 disease with reinfection. Computational Biology and Chemistry.

[bib8] BC centre for Disease Control (2009).

[bib9] Bertholom C. (2021). Réponse immunitaire associée au sars-cov-2. Option/Bio.

[bib10] Bocharov G.A., Romanyukha A.A. (1994). Mathematical model of antiviral immune response iii. influenza a virus infection. Journal of Theoretical Biology.

[bib11] Boianelli A., Nguyen Van K., Ebensen T., Schulze K., Wilk E., Sharma N. (2015). Modeling influenza virus infection: A roadmap for influenza research. Viruses.

[bib12] Bonilla F.A., Oettgen H.C. (2010). Adaptive immunity. Journal of Allergy and Clinical Immunology.

[bib13] Brock C.K., Wallin S.T., Ruiz O.E., Samms K.M., Mandal A., Sumner E.A. (2019). Stem cell proliferation is induced by apoptotic bodies from dying cells during epithelial tissue maintenance. Nature Communications.

[bib14] Cao P., Yan A.W.C., Heffernan J.M., Petrie S., Moss R.G., Carolan L.A. (2015). Innate immunity and the inter-exposure interval determine the dynamics of secondary influenza virus infection and explain observed viral hierarchies. PLoS Computational Biology.

[bib15] Carruthers J., Xu J., Finnie T.J.R., Hall I. (2022). A within-host model of sars-cov-2 infection. medRxiv.

[bib16] Chatterjee B., Singh Sandhu H., Dixit N.M. (2022). Modeling recapitulates the heterogeneous outcomes of sars-cov-2 infection and quantifies the differences in the innate immune and cd8 t-cell responses between patients experiencing mild and severe symptoms. PLoS Pathogens.

[bib17] Dan J.M., Mateus J., Kato Y., Hastie K.M., Yu E.D., Faliti C.E. (2021). Immunological memory to sars-cov-2 assessed for up to 8 months after infection. Science.

[bib18] de Carvalho Sales-Peres S.H., Azevedo-Silva L.J.de, Carolina Soares Bonato R., de Carvalho Sales-Peres M., Pinto A.C.da S., Santiago Junior J.F. (2020). Coronavirus (sars-cov-2) and the risk of obesity for critically illness and icu admitted: Meta-analysis of the epidemiological evidence. Obesity Research & Clinical Practice.

[bib19] Dempsey P.W., Vaidya S.A., Cheng G. (2003). The art of war: Innate and adaptive immune responses. Cellular and Molecular Life Sciences CMLS.

[bib20] Diekmann O., Heesterbeek J.A.P., Metz J.A.J. (1990). On the definition and the computation of the basic reproduction ratio r 0 in models for infectious diseases in heterogeneous populations. Journal of Mathematical Biology.

[bib21] Dogra P., Schiavone C., Wang Z., Ruiz-Ramírez J., Caserta S., Staquicini D.I. (2023). A modeling-based approach to optimize covid-19 vaccine dosing schedules for improved protection. JCI insight.

[bib22] Elaiw A.M., Agha AD Al, Azoz S.A., Ramadan E. (2022). Global analysis of within-host SARS-CoV-2/HIV coinfection model with latency. The European Physical Journal Plus.

[bib23] Elbaz I.M., Sohaly M.A., El-Metwally H. (2022). Modeling the stochastic within-host dynamics sars-cov-2 infection with discrete delay. Theory in Biosciences.

[bib24] Fatehi F., Bingham R.J., Dykeman E.C., Stockley P.G., Twarock R. (2021). Comparing antiviral strategies against covid-19 via multiscale within-host modelling. Royal Society Open Science.

[bib25] Geng Y., Wang Y. (2023). Stability and transmissibility of SARS-CoV-2 in the environment. Journal of Medical Virology.

[bib26] Ghosh I. (2021). Within host dynamics of sars-cov-2 in humans: Modeling immune responses and antiviral treatments. SN Computer Science.

[bib27] Gupta A., Marzook H., Ahmad F. (2023). Comorbidities and clinical complications associated with sars-cov-2 infection: An overview. Clinical and Experimental Medicine.

[bib28] Hancioglu B., Swigon D., Clermont G. (2007). A dynamical model of human immune response to influenza a virus infection. Journal of Theoretical Biology.

[bib29] Handel A., Antia R. (2008). A simple mathematical model helps to explain the immunodominance of cd8 t cells in influenza a virus infections. Journal of Virology.

[bib30] Hartley G.E., Edwards E.S.J., Aui P.M., Varese N., Stojanovic S., McMahon J. (2020). Rapid generation of durable b cell memory to sars-cov-2 spike and nucleocapsid proteins in covid-19 and convalescence. Science immunology.

[bib31] Hayden F.G., Fritz R., Lobo M.C., Alvord W., Strober W., Straus S.E. (1998). Local and systemic cytokine responses during experimental human influenza a virus infection. relation to symptom formation and host defense. Journal of Clinical Investigation.

[bib32] Jaber S., Conseil M., Coisel Y., Jung B., Chanques G. (2010).

[bib33] Julkunen I., Melén K., Nyqvist M., Pirhonen J., Sareneva T., Matikainen S. (2000). Inflammatory responses in influenza a virus infection. Vaccine.

[bib34] Kaech S.M., Wherry E.J., Ahmed R. (2002). Effector and memory t-cell differentiation: Implications for vaccine development. Nature Reviews Immunology.

[bib35] Kalia V., Sarkar S., Ahmed R. (2010). Cd8 t-cell memory differentiation during acute and chronic viral infections. Memory T cells.

[bib36] Kambayashi T., Assarsson E., Lukacher A.E., Ljunggren H.-G., Jensen P.E. (2003). Memory cd8+ t cells provide an early source of IFN-*γ*. The Journal of Immunology.

[bib37] Karachaliou M., Moncunill G., Espinosa A., Castaño-Vinyals G., Jiménez A., Vidal M. (2021). Infection induced sars-cov-2 seroprevalence and heterogeneity of antibody responses in a general population cohort study in catalonia Spain. Scientific Reports.

[bib38] Kim K.S., Ejima K., Iwanami S., Fujita Y., Ohashi H., Koizumi Y. (2021). A quantitative model used to compare within-host sars-cov-2, mers-cov, and sars-cov dynamics provides insights into the pathogenesis and treatment of sars-cov-2. PLoS Biology.

[bib39] Kono H., Rock K.L. (2008). How dying cells alert the immune system to danger. Nature Reviews Immunology.

[bib40] Lee H.Y., Topham D.J., Park S.Y., Hollenbaugh J., Treanor J., Mosmann T.R. (2009). Simulation and prediction of the adaptive immune response to influenza a virus infection. Journal of Virology.

[bib41] Li C., Xu J., Liu J., Zhou Y. (2020). The within-host viral kinetics of sars-cov-2. bioRxiv.

[bib42] Marchuk G.I., Petrov R.V., Romanyukha A.A., Bocharov G.A. (1991). Mathematical model of antiviral immune response. i. data analysis, generalized picture construction and parameters evaluation for hepatitis b. Journal of Theoretical Biology.

[bib43] Mazzoni A., Maggi L., Capone M., Vanni A., Spinicci M., Salvati L. (2021). Heterogeneous magnitude of immunological memory to sars-cov-2 in recovered individuals. Clinical & Translational Immunology.

[bib44] McDonagh M., Bell E.B. (1995). The survival and turnover of mature and immature cd8 t cells. Immunology.

[bib45] McMahon A., Robb N.C. (2020). Reinfection with sars-cov-2: Discrete sir (susceptible, infected, recovered) modeling using empirical infection data. JMIR Public Health Surveill.

[bib46] Mondal J., Samui P., Chatterjee A.N. (2022). Dynamical demeanour of sars-cov-2 virus undergoing immune response mechanism in covid-19 pandemic. The European Physical Journal - Special Topics.

[bib47] Natoli G., Ostuni R. (2019). Adaptation and memory in immune responses. Nature Immunology.

[bib48] Netea M.G., Domínguez-Andrés J., Barreiro L.B., Chavakis T., Divangahi M., Fuchs E. (2020). Defining trained immunity and its role in health and disease. Nature Reviews Immunology.

[bib49] Nicolet B.P., Guislain A., van Alphen F.P.J., Gomez-Eerland R., Schumacher T.N.M., van den Biggelaar M. (2020). Cd29 identifies ifn-*γ*–producing human cd8+ t cells with an increased cytotoxic potential. Proceedings of the National Academy of Sciences.

[bib50] Park J.-ha, Kotani T., Konno T., Setiawan J., Kitamura Y., Imada S. (2016). Promotion of intestinal epithelial cell turnover by commensal bacteria: Role of short-chain fatty acids. PLoS One.

[bib51] Perasso A. (2018). An introduction to the basic reproduction number in mathematical epidemiology. ESAIM: Proceedings and Surveys.

[bib52] Peters-Hall J.R., Min J., Tedone E., Sho S., Siteni S., Mender I. (2018). Human lung epithelial cells divide> 200 population doublings without engaging a telomere maintenance mechanism. bioRxiv.

[bib53] Ryu G., Shin H.-W. (2021). Sars-cov-2 infection of airway epithelial cells. Immune network.

[bib54] Sadria M., Layton A.T. (2021). Modeling within-host SARS-CoV-2 infection dynamics and potential treatments. Viruses.

[bib55] Schuh L., Markov P.V., Veliov V.M., Stilianakis N.I. (2024). A mathematical model for the within-host (re) infection dynamics of sars-cov-2. Mathematical Biosciences.

[bib56] Sette A., Crotty S. (2022). Immunological memory to sars-cov-2 infection and covid-19 vaccines. Immunological Reviews.

[bib57] Smith A.M., Perelson A.S. (2011). Influenza a virus infection kinetics: Quantitative data and models. Wiley Interdisciplinary Reviews: Systems Biology and Medicine.

[bib58] Tamura S.-ichi, Tanimoto T., Kurata T. (2005). Mechanisms of broad cross-protection provided by influenza virus infection and their application to vaccines. Japanese Journal of Infectious Diseases.

[bib59] Tang X., Wu C., Xiang L., Song Y., Yao X., Wu X. (2020). On the origin and continuing evolution of sars-cov-2. National Science Review.

[bib60] Ul Haq I., Yavuz M., Ali N., Ali A. (2022). A sars-cov-2 fractional-order mathematical model via the modified euler method. Mathematical and Computational Applications.

[bib61] Ursin R.L., Shapiro J.R., Klein S.L. (2020). Sex-biased immune responses following sars-cov-2 infection. Trends in Microbiology.

[bib62] Van de Sandt C.E., Kreijtz J.H.C.M., Rimmelzwaan G.F. (2012). Evasion of influenza a viruses from innate and adaptive immune responses. Viruses.

[bib63] Van den Driessche P., Watmough J. (2002). Reproduction numbers and sub-threshold endemic equilibria for compartmental models of disease transmission. Mathematical Biosciences.

[bib64] Xu W., Banchereau J. (2014). The antigen presenting cells instruct plasma cell differentiation. Frontiers in Immunology.

[bib65] Xu J., Carruthers J., Finnie T., Hall I. (2023). Simplified within-host and dose–response models of sars-cov-2. Journal of Theoretical Biology.

[bib66] Xu Z., Wei D., Zhang H., Demongeot J. (2023). A novel mathematical model that predicts the protection time of sars-cov-2 antibodies. Viruses.

[bib67] Yan A.W.C., Zaloumis S.G., Simpson J.A., McCaw J.M. (2019). Sequential infection experiments for quantifying innate and adaptive immunity during influenza infection. PLoS Computational Biology.

[bib68] Zarnitsyna V.I., Handel A., McMaster S.R., Hayward S.L., Kohlmeier J.E., Antia R. (2016). Mathematical model reveals the role of memory cd8 t cell populations in recall responses to influenza. Frontiers in Immunology.

[bib69] Zhou P., Yang X.-L., Wang X.-G., Hu B., Zhang L., Zhang W. (2020). A pneumonia outbreak associated with a new coronavirus of probable bat origin. Nature.

[bib70] Zuiani A., Wesemann D.R. (2022). Antibody dynamics and durability in coronavirus disease-19. Clinics in Laboratory Medicine.

